# Drug repositioning: a machine-learning approach through data integration

**DOI:** 10.1186/1758-2946-5-30

**Published:** 2013-06-22

**Authors:** Francesco Napolitano, Yan Zhao, Vânia M Moreira, Roberto Tagliaferri, Juha Kere, Mauro D’Amato, Dario Greco

**Affiliations:** 1 Department of Computer Science, University of Salerno, Salerno, Italy; 2 Telethon Institute of Genetics and Medicine (TIGEM), Naples, Italy; 3 Research Unit of Molecular Medicine, University of Helsinki, Helsinki, Finland; 4 Division of Pharmaceutical Chemistry, Faculty of Pharmacy, University of Helsinki, Helsinki, Finland; 5 Department of Biosciences and Nutrition, Karolinska Institutet, Stockholm, Sweden

**Keywords:** Drug repositioning, Connectivity map, CMap, ATC code, Mode of action, Machine learning, SVM, Integrative genomics, SMILES, Anthelmintics, Antineoplastic, Oxamniquine, Niclosamide

## Abstract

Existing computational methods for drug repositioning either rely only on the gene expression response of cell lines after treatment, or on drug-to-disease relationships, merging several information levels. However, the noisy nature of the gene expression and the scarcity of genomic data for many diseases are important limitations to such approaches. Here we focused on a drug-centered approach by predicting the therapeutic class of FDA-approved compounds, not considering data concerning the diseases. We propose a novel computational approach to predict drug repositioning based on state-of-the-art machine-learning algorithms. We have integrated multiple layers of information: i) on the distances of the drugs based on how similar are their chemical structures, ii) on how close are their targets within the protein-protein interaction network, and iii) on how correlated are the gene expression patterns after treatment. Our classifier reaches high accuracy levels (78%), allowing us to re-interpret the top misclassifications as re-classifications, after rigorous statistical evaluation. Efficient drug repurposing has the potential to significantly impact the whole field of drug development. The results presented here can significantly accelerate the translation into the clinics of known compounds for novel therapeutic uses.

## Background

Despite the enormous increase of financial investments in pharmaceutical R&D, the number of newly approved drugs has greatly diminished during the past decade [1]. Finding new uses for approved drugs has consequently become a major alternative strategy for the pharma industry. This practice, usually referred to as drug repositioning, is highly attractive because of its potential to speed up the process of drug development, hence reducing costs in addition to providing new treatments for unmet medical needs [2]. In this regard, compounds that have passed through phases II or III in the drug discovery pipeline but never made it to the market due to efficacy issues bear great potential for drug repositioning approaches. Successful drug repositioning requires that a known drug has a positive impact on a different disease, but its highest value resides in that its use for the novel indication surpasses the currently available therapeutic options for that condition. Experimental approaches to drug repositioning generally involve high-throughput assays where libraries of approved compounds are tested against biological targets of interest. The effects of a large number of Food and Drug Administration (FDA)-approved compounds on gene expression have been measured on several cultured human cell lines (the Connectivity Map, CMap) [3], and these information has been used to investigate similarities between drugs mechanisms of action [4]. Further, the CMap data has been systematically re-analyzed in search of differential expression patterns of the genes encoding the drug targets [5]. Computational approaches more specifically aimed at drug repositioning have been designed to find correlations between disease-associated and drug-associated expression signatures under the assumption that an effective drug should be able to counterbalance the perturbations caused by a disease. Remarkably, this kind of approach has already led to the identification and experimental validation of novel therapeutic indications for the antiepileptic topiramate in inflammatory bowel disease (IBD) [6]. Genetic risk effects associated with druggable genes in complex diseases have also been considered in order to attempt drug repositioning [7]. Finally, more comprehensive methods that take into account chemical, molecular and biological aspects of the drug-disease interactions have also been recently proposed [8]. Although the above studies have demonstrated that computational approaches to drug repositioning are feasible, there are still large margins for improvement. For instance, deriving drug repositioning from drug-disease interactions alone can be difficult due to the complexity, variability and sparsity of data currently available for the diseases, and to the intrinsic nature of publicly available gene expression data, which derive from patients already treated with other drugs in most of the cases. In order to overcome such limitations, in this study we have decided to establish a methodological approach focusing primarily on drug characteristics. Aiming at enhancing the predictive power of the available computational methods, we have developed a novel approach based on machine-learning classification algorithms, where mismatches between known and predicted drug classifications are purposely interpreted as potential alternative therapeutic indications. We have studied 410 drugs by integrating different layers of information based on their similarities, including their chemical structures, molecular targets and induced gene expression signatures.

## Results and discussion

### Computational pipeline

**Figure 1:**
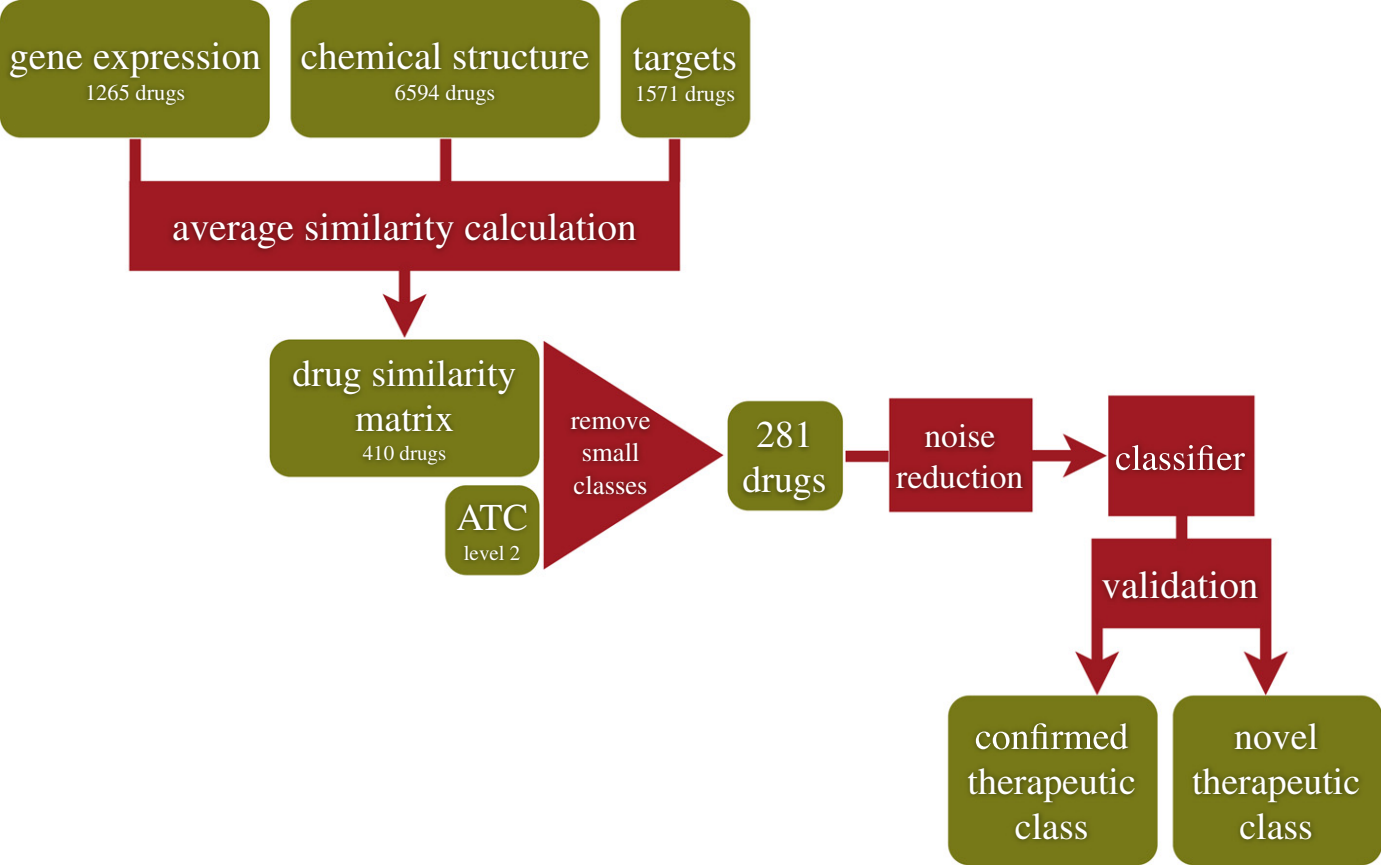
**Flowchart of the analysis.** Green boxes indicate data, red boxes indicate processes.

**Figure 2:**
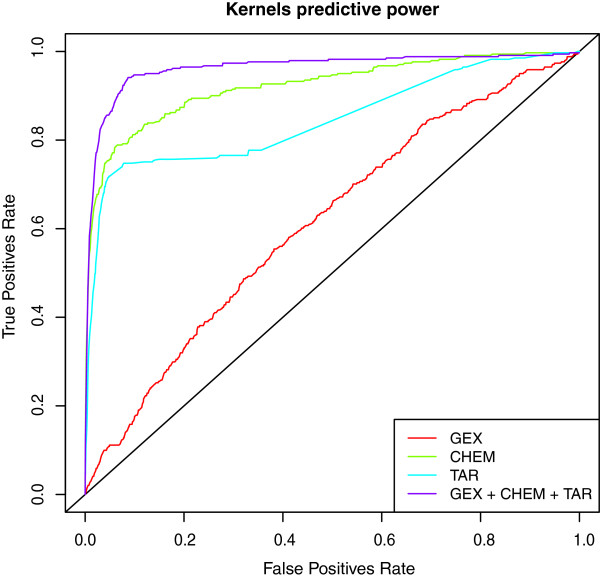
**Classification performance and data integration.** Receiving Operator Curve for the three separated kernels (GEX: gene expression; CHEM: chemical structure; TAR: molecular targets) and the final joint kernel in respect with ATC classes.

We have integrated different techniques and data sources in order to build a classifier whose outcome is a therapeutic class for a given drug. The steps of our computational strategy are summarized in Figure 1. We have first re-analyzed the CMap gene expression data using state-of-the-art methods for probe annotation and normalization. The drug-drug similarities for the gene expression layer have been based on the ranks of the genes in each drug-induced expression profile and their associated p-values. Next, pairwise similarities for the molecular structures have been assessed by computing the distances between the corresponding binary fingerprints. Finally, target-based similarities have been obtained by taking into account known common targets and their distances across the global human protein-protein interaction network. Subsequently, we have combined the drug similarities into a single information layer used to train a multi-class SVM (Support Vector Machine) classifier [9]. Receiver Operating Characteristic (ROC) curves show that integrating information coming from different sources into a single kernel improves the performance of the corresponding classifier (Figure 2). This has also been confirmed by testing the performance of the classifiers built on a single information layer. Similarity (kernel) matrices have the advantage of being directly comparable even when computed from highly heterogeneous data. However, our kernels, designed to weigh information according to what is considered a priori to be relevant (molecular structure features in the fingerprints, differentially expressed genes in profiles, known protein-protein interactions), have shown poor results when used to train kernel classifiers. Conversely, proper projections of the data by using Classical Multidimensional Scaling (cMDS, or Principal Coordinate Analysis [10], see Section “Materials and Methods”) and subsequent computation of a classical kernel (Gaussian) has allowed higher classification rates, thus providing an effective way to transform a kernel directly built from biological data to another that is technically efficient. The therapeutic class of each drug has been extracted from the Anatomical Therapeutic Chemical classification (ATC, see Section “Materials and Methods”), as defined by the World Health Organization (WHO). By using the ATC second level (*therapeutic subgroup*) and the classes with at least 8 drugs, with the best kernel, we have applied random sub-sampling and re-training iterations both to improve classification performance and to reduce over-fitting, thus contributing to the final 78% accuracy, and producing reliable hints for repositioning. The final classification for each drug has been thus obtained by choosing the most frequently predicted ATC code. On the other hand, hints for repositioning have been obtained as the most frequent misclassifications. Thus, a classifier is built in order to obtain knowledge about known samples, as opposed to predict classes for new ones. Moreover, the inverted use of the classifier results, where correct classifications are used to assess the reliability of the misclassifications, puts automatic classifiers in a new perspective that could provide more interesting applications in the future.

Our computational strategy of drug repositioning crosses previous work at different levels. A recent study [11] has tackled our same task of predicting ATC codes by collecting chemical data for 3,883 drugs. Although our study is focused on a more specific level of ATC codes (level 2 as opposed to level 1) and includes a smaller database of drugs (a sizeable portion of drugs are not in CMap), our method provides higher classification performance (78% as compared to 73%). The predictive power of gene expression alone with respect to ATC codes has been also investigated in two related studies [4,5]. While focusing on drugs mechanisms of action, the first study has detected scarce correlation between similarities obtained through the gene expression profiles and those based on ATC codes. The second study has shown how this correlation could be improved by alternative data processing strategies. However, in both cases no attempt has been made at directly predicting ATC codes. More recently, an approach based on comparing multi-layered drug-drug and drug-disease similarities has been proposed to produce possible treatment predictions [8]. The results have been validated through Area Under the ROC Curve (AUC). From a methodological point of view, the classification accuracy of our approach cannot be directly compared with such score, which is meant to test edge predictions, as opposed to class predictions. From a methodological point of view, the main novelty of our work resides in the development of a data integration framework for efficiently predicting drugs ATC codes and in its use as a tool for drug repositioning.

### Drug repositioning

**Figure 3:**
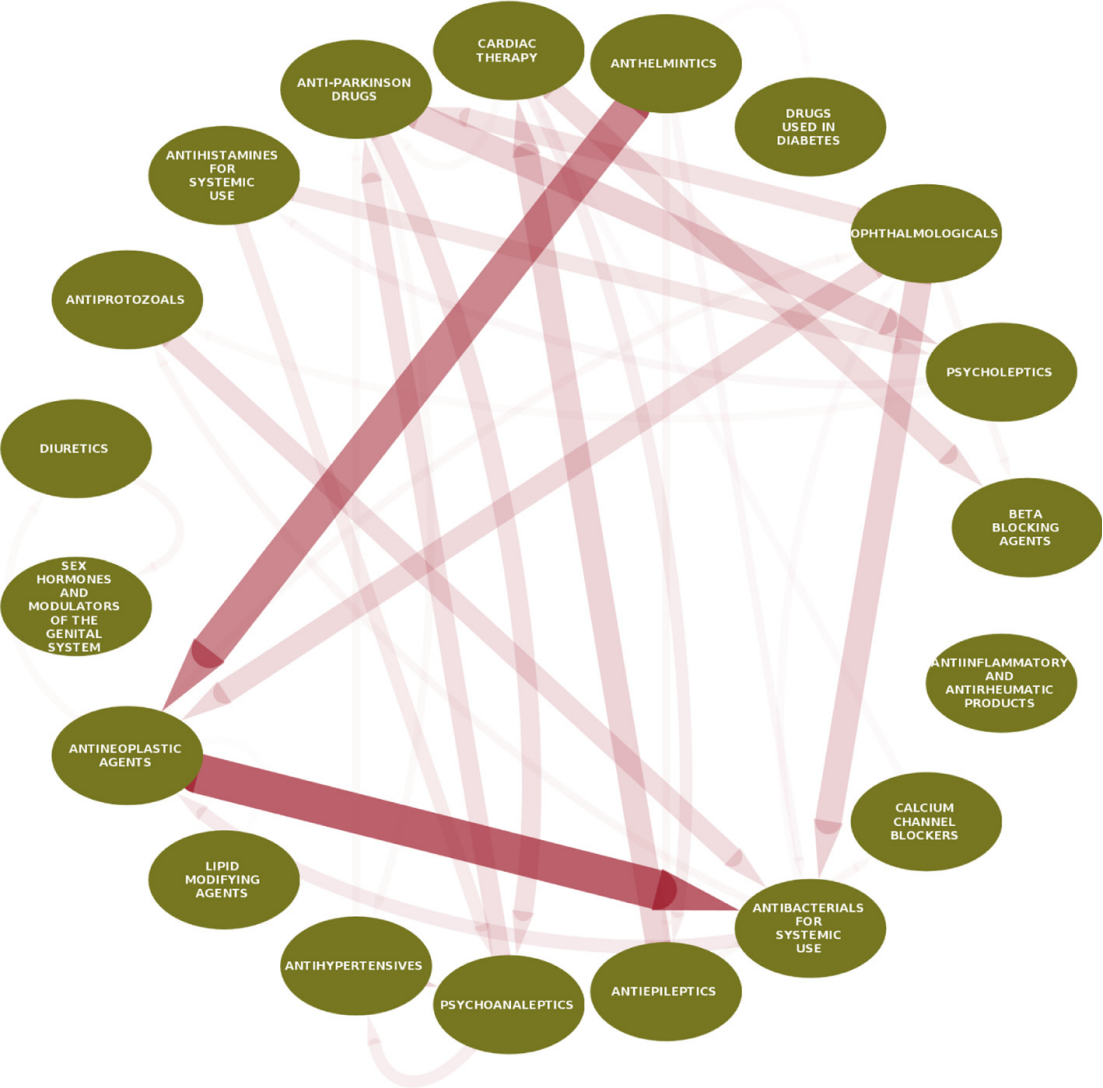
**Repositioning overview.** Direction of the arrows represent direction of repositioning from one ATC class to another. Thickness and opacity of the edges represent a score-weighted sum of the reclassification events.

Figure 3 shows the main trends of repositioning highlighting that, among our selected drug portofolio, the repositioning of antihelmintics to antineoplasic agents and of antineoplasic agents to antibacterials of systemic use were the most frequent drug reclassifications. Table 1 highlights the 12 top scoring drug repositionings identified by our model (presented in full in Additional file 1). We have found that our model correctly assigned the corresponding activity of the ophthalmologicals levobunolol and sulfacetamide to beta-blocking agents and antibacterials, respectively. In addition, it has accurately underlined drugs known structural similarities, such as the beta-adrenergic agonist dobutamine reclassified from cardiac therapy to beta blocking agents, and the antihelmintic ivermectin reclassified as an antibacterial. It is known that, despite being structurally similar to macrolide antibiotics and antifungal macrocyclic polyenes, irvemectin is actually devoid of antibacterial or antifungal activities [12]. Inspection of our results also indicates that our method has accurately predicted very plausible alternative therapeutic classes for known drugs. Antihistamines, known to have antipsychotic effects (chlorphenamine, thiethylperazine) or currently in use for their antipsychotic properties (hydroxyzine), were reclassified as psychoanaleptics/psycholeptics. Finally, the antiepileptic carbamazepine, known to have cardiovascular side effects, has been reclassified for cardiac therapy, and the diuretic spirolonactone, which has known anti-androgenic effects, has been repositioned in the class of sex hormones and modulators of the genital system. The repositioning of antihelmintics to antineoplasic agents is consistent with the fact that some antihelmintic drugs included in this study (benzimidazole antihelmintics) interfere with microtubule synthesis in the parasites and could have the potential to cause mitotic arrest in tumor cells. Anticancer properties related to microtubule disruption have already been reported for mebendazole and albendazole, which has inclusively been studied on Phase I clinical trials for patients with advanced cancer [13]. Mebendazole has been reported to show survival benefit in two preclinical models of glioblastoma multiforme, and to induce apoptosis of several cancer cell lines including melanoma, human adrenocortical carcinoma, and non-small cell lung cancer [14-18]. Praziquantel, which is chemically different from the benzimidazole antihelmintics, has been however best repositioned by our model as an antiepileptic, most likely due to its effects on calcium homeostasis. Of note, the antihelmintics niclosamide and oxamniquine have been also repositioned as anticancer agents. In agreement to our computational prediction, niclosamide has been recently shown multiple anticancer effects in tumors of the ovary and colon, and also in leukemia and myeloma [19-24]. Some of its molecular targets have been disclosed and include, among others, the Wnt/Frizzled 1 [25], the mammalian target of rapamycin complex 1 (mTOR) [26], and the signal transducer and activator of transcription 3 (STAT 3) [27] signaling pathways. Interestingly, oxamniquine exerts its antihelmintic effects by causing paralysis and contraction of the worms after interference with their DNA [28], a mechanism that could also account for its potential activity in cancer. Thus, not only our model has predicted the repositioning of several antihelmintics to anticancer agents in line with most recent literature, but has also suggested that a systematic investigation of this therapeutic class may disclose important information that could be of therapeutic use for anticancer treatment and/or drug discovery. The significance of the direct repositioning of antineoplasic drugs as systemic antibacterials is however more difficult to extrapolate because most of these drugs do not offer advantages to the antimicrobials in current use due to toxicity issues. Nonetheless, gefitinib, a more selective chemotherapeutic agent, presents the highest score for reposition as antibacterial. To the best of our knowledge, there is yet no prior evidence or related supportive information concerning this finding, which could pave the way for the development of a novel class of antibacterials.

**Table 1:** Top drug repositioning predictions

**Drug name**	**Original ATC therapeutic class**	**Predicted ATC therapeutic class**
Carbamazepine	Antiepileptics (N03)	Cardiac therapy (C01)
Chlorphenamine	Antihistamines for systemic use (R06)	Psychoanaleptics (N06)
Dobutamine	Cardiac therapy (C01)	Beta blocking agents (C07)
Gefitinib	Antineoplastic agents (L01)	Antibacterials for systemic use (J01)
Hydroxyzine	Psycholeptics (N05)	Antihistamines for systemic use (R06)
Ivermectin	Anthelmintics (P02)	Antibacterials for systemic use (J01)
Levobunolol	Ophthalmologicals (S01)	Beta blocking agents (C07)
Niclosamide	Anthelmintics (P02)	Antineoplastic agents (L01)
Oxamniquine	Anthelmintics (P02)	Antineoplastic agents (L01)
Spironolactone	Diuretics (C03)	Sex hormones and modulators of the genital system (G03)
Sulfacetamide	Ophthalmologicals (S01)	Antibacterials for systemic use (J01)
Thiethylperazine	Antihistamines for systemic use (R06)	Psycholeptics (N05)

The top 12 repositioned drugs (classification score =1) are shown in rows. The drug name, the original and the predicted therapeutic class are reported. The level 2 ATC codes are also reported in brackets.

## Conclusions

In summary, we report a novel computational approach to predict drug repositioning based on a machine-learning algorithm and data integration. The novelty of our approach relies on the purposeful interpretation of classification mismatches as genuine reclassifications opportunities. Our procedure also gains from integrating different layers of information and maximizing their efficacy through computational procedures based on dimensionality reduction. Our results showed high accuracy levels, which were consistent with several literature reports. We believe our work offers new directions towards repositioning of known drugs and also for the development of novel drug discovery programs.

## Methods/Experimental

### Microarray data processing

**Figure 4:**
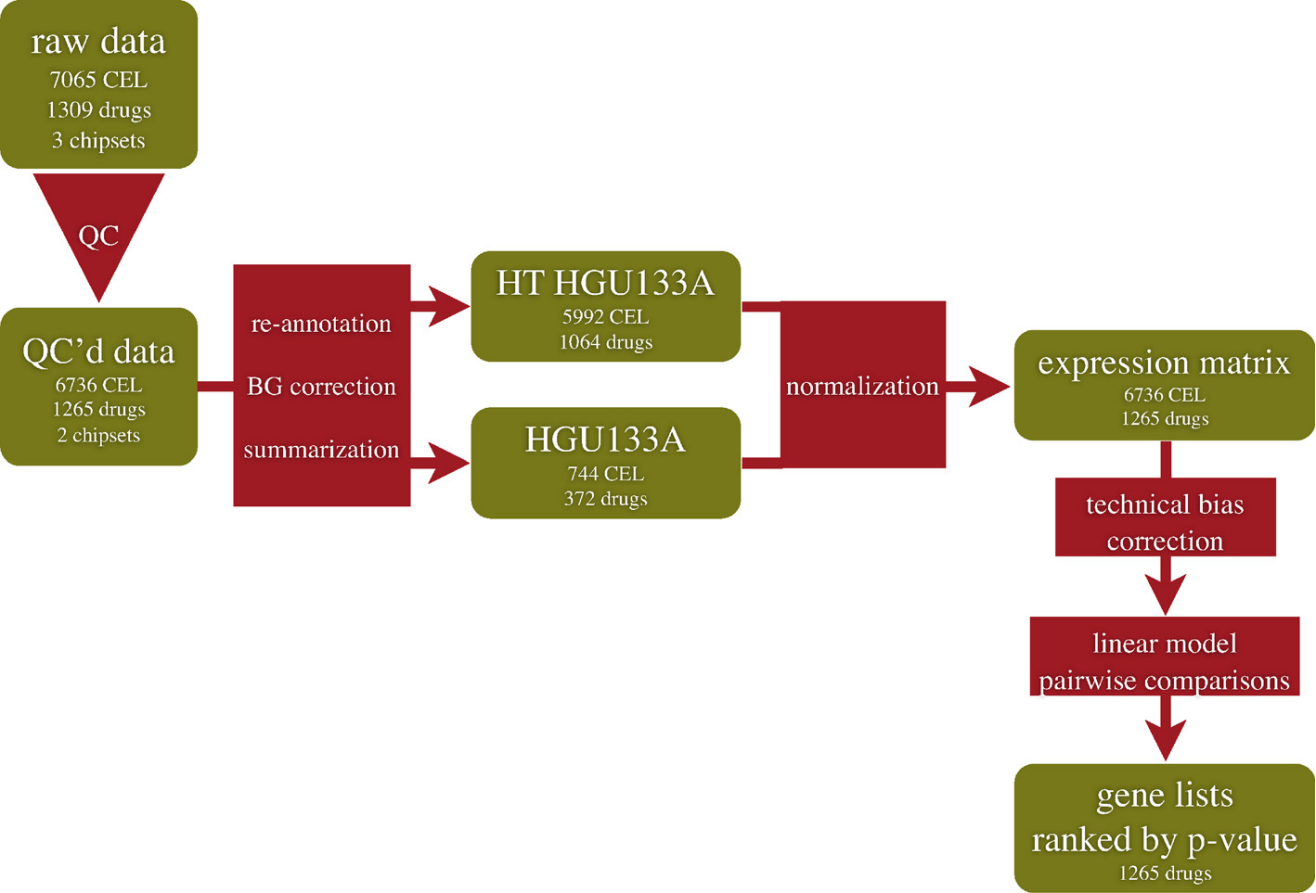
**Flowchart of the CMap gene expression data analysis.** Green boxes indicate data, red boxes indicate processes.

The processing pipeline for the Microarray data is illustrated in Figure 4. A total of 7056 Affymetrix GeneChip raw data files (.CEL files) belonging to two chipsets (HG-U133A and HTHG-U133A) were collected from the Connectivity Map (CMap) [3] website and imported into R v. 2.12.1 [29]. Raw data files were quality checked using the R package affy v.1.32.0 [30] and affyQCReport v.1.32.0 [31] to exclude the poor quality data points, resulting in a set of usable 6736 CEL files. The probes of each chipset were re-annotated according to NCBI Entrez Gene database [32]. For this, the CDF packages v.14.1.0 were downloaded from brainarray website [33]. The background estimation and the probe summarization were done on the raw data from each chipset separately according to the RMA algorithm [34]. The two data matrices were then combined for 12139 common probe-sets obtaining an expression matrix of dimensions 12139×6736. Consequently, this matrix was normalized with the quantile method. Next, the ComBat algorithm was used to estimate and remove the technical bias (array type, scanner and vehicle) from the normalized data matrix [35]. Linear models followed by moderated t-test statistic were used to compute the p-values and the fold-changes in each drug-control pairs, by the limma package v.3.10.0 [36].

### Drug similarities

The Jaccard Index *JI*, the Cosine Similarity *CS* and the Dice Coefficient *DC* similarity measurements were used to calculate the similarity between drugs based on their molecular structure in form of fingerprint of simplified molecular-input line-entry specification (SMILES [37]) retrieved for 6594 small molecules from the DrugBank database [38] and processed by the package rcdk v.3.1.3 [39]. Let D be as set of drugs {*D*_1_,*D*_2_,…,*D*_*n*_}. Let $ℬ=\{{ℬ}_{1},{ℬ}_{2},\ldots ,{ℬ}_{n}\}$ be such that ${ℬ}_{i}$ is the binary fingerprint representation of the drug *D*_*i*_, *i*∈{1,…*n*}. Note that binary vectors can be seen as representations of sets, where included elements are indicated as 1-s in the vector. With abuse of notation, here we use ${ℬ}_{i}$ as its corresponding set. We defined the dissimilairty measure between any two drugs $D_{i},\;D_{j}\in D$ as:

$$\begin{array}{c} K^{\mathrm{CHEM}}(D_{i},D_{j})=1-\frac{\text{JI}({ℬ}_{i},{ℬ}_{j})+\text{CS}({ℬ}_{i},{ℬ}_{j})+\text{DC}({ℬ}_{i},{ℬ}_{j})}{3} \end{array}$$

The same measure was used to compute drug similarities based on molecular targets, obtained for 1571 drugs from the DrugBank database. Given a drug $D_{i}\in D$, let $T_{i}$ be the set of targets associated with the drug *D*_*i*_ in DrugBank. Let ${\hat{K}}^{\text{CHEM}}$ be defined like *K*^CHEM^, but with ${ℬ}_{i}=T_{i},\;i\in \{1,\ldots ,n\}$. We used $K^{\mathrm{TA}R^{\prime }}(D_{i},D_{j})={\hat{K}}^{\text{CHEM}}(D_{i},D_{j})$ as the dissimilarity value between two drugs *D*_*i*_ and *D*_*j*_. To cope with the scarce granularity of this measure, we also defined a finer dissimilarity measure $K^{\text{TA}R^{\prime \prime }}$ based on on the PPI database [40,41] as follows. Let *P*_*i*,*j*_ be the set of shortest paths from each target in *T*_*i*_ to each target in *T*_*j*_ according to the PPI network. We used the range-normalized length of the shortest among the paths in *P*_*i*,*j*_ as the dissimilarity value between drug *i* and drug *j*. As final molecular target dissimilarity measure we used:

$$K^{\text{TAR}}(D_{i},D_{j})=\frac{K^{\text{TA}R^{\prime }}(D_{i},D_{j})+K^{\text{TA}R^{\prime \prime }}(D_{i},D_{j})}{2}$$

Finally, we used a weighted Spearman’s Footrule (WSF) as a drug dissimilarity measure based on gene expression profiles from the Cmap as ranked and weighted according to their p-values. Given the gene expression profiles associated with two drugs *D*_*i*_ and *D*_*j*_, we ranked the genes in ascending order of their signed p-values, where the sign is given by the opposite of the sign of their fold change. This way, top-ranking genes were those over-expressed with low p-values and bottom-ranking genes were those under-expressed with low p-values. Genes associated with high p-values tend to stay in the middle of the ranked lists. Let’s define *R*_*D*_(*g*) as such rank for the gene *g* in the expression profile of drug *D*. Analogously, let *W*_*D*_(*g*) be 1 minus the p-value of the gene *g* in the expression profile of drug *D*. The gene expression profile dissimilarity measure between drug *i* nd *j* was then defined as:

$$\begin{array}{c} \;K^{\text{GEX}}(D_{i},D_{j})=N\left(\underset{g}{\sum }\left|R_{i}(g)-R_{j}(g)\right|\left[\frac{W_{i}(g)+W_{j}(g)}{2}\right]\right) \end{array}$$

where, in order to simplify notation, we used N to indicate range normalization.

### Data integration

Data were collected in the form of dissimilarity matrices in order to easily integrate information over the three datasets (gene expression, chemical structure and molecular targets). The three databases had 410 drugs in common. Let D be the set of such drugs. We simply defined the joint kernel matrix *K* as:

$$\begin{array}{rl} \forall \left(i,j\right) & :\{D_{i},D_{j}\}\subset D,\;{\hat{K}}_{i,j}=\\ & \frac{K^{\mathrm{GEX}}(D_{i},D_{j})+K^{\text{TAR}}(D_{i},D_{j})+K^{\mathrm{CHEM}}(D_{i},D_{j})}{3} \end{array}$$

$\hat{K}$ was a 410×410 symmetric matrix representing our training set for the prediction of level 2 ATC codes. Since the number of different ATC codes at level 2 is large compared with the number of drugs in our dataset, a number of classes appeared empty or highly under-represented at that level. For this reason we removed all the drugs falling into ATC classes with less than 8 exemplars, obtaining a final 281×281 kernel *K*. We made this choice on the basis of a required lower bound on the classification performance of 75%, which is verified a posteriori with a simpler version (without bootstrap) of the approach described in the next Section.

### Noise reduction and classification

Let *K* be the 281×281 kernel as defined in the previous Section. The ATC classification codes for such drugs were retrieved from the World Health Organization (WHO). In this system, the active compounds are grouped according to relevant pharmacological and clinical properties. The classification is organized in five hierarchical levels: the first level describes the anatomical site where the compound is active; the second level refers to pharmacological/therapeutic subgroups; the third and fourth levels define chemical/pharmacological/therapeutic subgroups. In the fifth level, the individual substances are identified. We used ATC codes (at second level: *therapeutic subgroup*) as targets for our classifier.

In order to maximize the efficiency of this kernel, we exploited Classical Multidimensional Scaling (cMDS, or Principal Coordinate Analysis [10]) to search for an optimal Euclidean embedding of the 281 drugs in the following way. The number of eigenvalues larger than 0 was 241. Let *M*_*i*_∀*i*∈{1,…,241} be the 241×*i* matrix representing the cMDS projection of *K* into the sub-space spanned by the first *i* Principal Components of *K*. Let *e*(*M*_*i*_) be the classification error of a Support Vector Machine (native Multiclass SVM [42] using Gaussian kernel) on *M*_*i*_ by 6-fold cross validation. Cross-validation was used to reduce the risk of over-fitting the data during the assessment of the model. K-fold is chosen because of the low number of samples. Note that in our context the minimization of the validation error is only needed to obtain reliable classifications for the given data and not to assess the performance of the classifier on new data.

**Figure 5:**
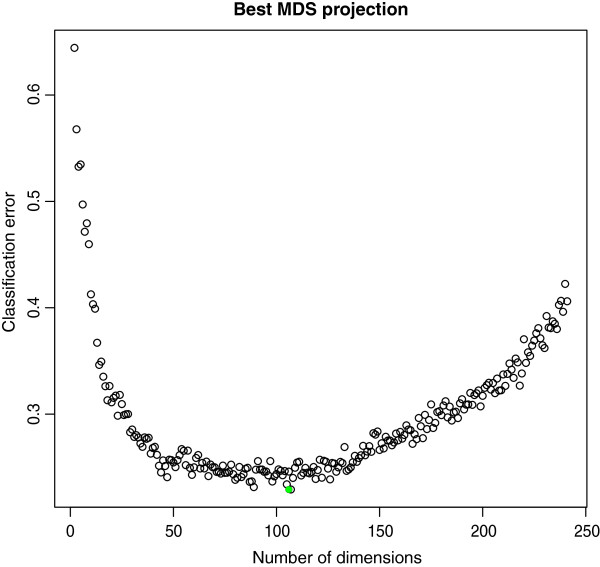
**Kernel noise reduction through MDS projections.** Values on the *x* axis correspond to SVM classifiers trained with kernel *K* (see text) as projected into the subspace spanned by its first *x* Principal Components. The *y* axis reports the corresponding 6-fold cross validation error. The green dot indicates minimum error.

The validation error *e*(*M*_*i*_)∀*i*∈{1,…,241}, when plotted versus the number of dimensions, suggests the existence of a hypothetical smooth latent function between the two variables (See Figure 5). We choose arg *i*∈{1,…,241}*mine*(*M*_*i*_)=107 as a reasonable approximation of the theoric minimum of such hypothetical function. On the 281 drugs at ATC level 2 projected on the 107-dimensional MDS subspace, using 10,000 bootstrap iterations with 10% holdout (see also next Section), we reached a final classification performance of 78%.

### From misclassifications to repositioning

For each drug we assigned a probability distribution to the outcome of the classier basing on the frequencies collected during bootstrap. Let $D_{i},\;i\in \{1,2,\ldots ,m\}$ be the subsets of D obtained during bootstrap iteration *i*. Each $D_{i}$ contained 90% of the drugs of D and was used to train an SVM. Different sets induce different predictions for each drug in general, with the level of variability depending on the sensitivity of each data point to perturbations in the overall distribution. Given a drug $D_{j}\in D$, and *m* predictions *P*_*j*_={*p*_1_, …,*p*_*m*_}, *p*_*i*_∈{1,…,*c*}, where *c* is the number of classes, we defined the *repositioning scoreS*_*i*,*j*_ of each prediction *p*_*i*_ for the drug *D*_*j*_ as the frequency of *p*_*i*_ in *P*_*j*_. While the final prediction for the drug *D*_*j*_ was assumed to be the most frequent one (which gives overall 78% accuracy as stated in the previous Section), we used all the other predictions as repositioning suggestions and their *S*_*i*,*j*_ as a corresponding index of reliability. By definition, *S*_*i*,*j*_∈[0,1], with higher values indicating higher reliability. Table 1 reports all the obtained misclassifications having *S*_*i*,*j*_=1.

## Competing interests

The authors declare that they have no competing interests.

## Authors’ contributions

DG and MDA conceived the study; FN, YZ and DG designed and performed the computational pipeline; R.T. supervised the machine learning and pattern recognition aspects; F.N., Y.Z., V.M.M., R.T., J.K., M.D.A. and D.G. wrote the manuscript. All authors read and approved the final manuscript.

## Supplementary Material

Additional file 1 Complete list of the drug repositioning predictions Click here for file
