# Supplementary material for: Drug repositioning: a machine-learning approach through data integration
Source: J Cheminform. 2013 Jun 22;5:30. doi: 10.1186/1758-2946-5-30 (PMC3704944; doi:10.1186/1758-2946-5-30)
Supplement: Additional file 1 — Complete list of the drug repositioning predictions [file 1758-2946-5-30-S1.pdf]

Drug Name	original ATC therapeutic class	predicted ATC therapeutic class	Repositioned	Score
carbamazepine	ANTIEPILEPTICS (N03)	CARDIAC THERAPY (C01)	Y	1
chlorphenamine	ANTIHISTAMINES FOR SYSTEMIC USE (R06)	PSYCHOANALEPTICS (N06)	Y	1
dobutamine	CARDIAC THERAPY (C01)	BETA BLOCKING AGENTS (C07)	Y	1
gefitinib	ANTINEOPLASTIC AGENTS (L01)	ANTIBACTERIALS FOR SYSTEMIC USE (J01)	Y	1
hydroxyzine	PSYCHOLEPTICS (N05)	ANTIHISTAMINES FOR SYSTEMIC USE (R06)	Y	1
ivermectin	ANTHELMINTICS (P02)	ANTIBACTERIALS FOR SYSTEMIC USE (J01)	Y	1
levobunolol	OPHTHALMOLOGICALS (S01)	BETA BLOCKING AGENTS (C07)	Y	1
niclosamide	ANTHELMINTICS (P02)	ANTINEOPLASTIC AGENTS (L01)	Y	1
oxamniquine	ANTHELMINTICS (P02)	ANTINEOPLASTIC AGENTS (L01)	Y	1
spironolactone	DIURETICS (C03)	SEX HORMONES AND MODULATORS OF THE GENITAL SYSTEM (G03)	Y	1
sulfacetamide	OPHTHALMOLOGICALS (S01)	ANTIBACTERIALS FOR SYSTEMIC USE (J01)	Y	1
thiethylperazine	ANTIHISTAMINES FOR SYSTEMIC USE (R06)	PSYCHOLEPTICS (N05)	Y	1
acebutolol	BETA BLOCKING AGENTS (C07)	BETA BLOCKING AGENTS (C07)	N	1
acepromazine	PSYCHOLEPTICS (N05)	PSYCHOLEPTICS (N05)	N	1
acetazolamide	OPHTHALMOLOGICALS (S01)	OPHTHALMOLOGICALS (S01)	N	1
alprenolol	BETA BLOCKING AGENTS (C07)	BETA BLOCKING AGENTS (C07)	N	1
amitriptyline	PSYCHOANALEPTICS (N06)	PSYCHOANALEPTICS (N06)	N	1
amoxicillin	ANTIBACTERIALS FOR SYSTEMIC USE (J01)	ANTIBACTERIALS FOR SYSTEMIC USE (J01)	N	1
astemizole	ANTIHISTAMINES FOR SYSTEMIC USE (R06)	ANTIHISTAMINES FOR SYSTEMIC USE (R06)	N	1
azlocillin	ANTIBACTERIALS FOR SYSTEMIC USE (J01)	ANTIBACTERIALS FOR SYSTEMIC USE (J01)	N	1
aztreonam	ANTIBACTERIALS FOR SYSTEMIC USE (J01)	ANTIBACTERIALS FOR SYSTEMIC USE (J01)	N	1
bacampicillin	ANTIBACTERIALS FOR SYSTEMIC USE (J01)	ANTIBACTERIALS FOR SYSTEMIC USE (J01)	N	1
bisoprolol	BETA BLOCKING AGENTS (C07)	BETA BLOCKING AGENTS (C07)	N	1
brinzolamide	OPHTHALMOLOGICALS (S01)	OPHTHALMOLOGICALS (S01)	N	1
bumetanide	DIURETICS (C03)	DIURETICS (C03)	N	1
carbinoxamine	ANTIHISTAMINES FOR SYSTEMIC USE (R06)	ANTIHISTAMINES FOR SYSTEMIC USE (R06)	N	1
carmustine	ANTINEOPLASTIC AGENTS (L01)	ANTINEOPLASTIC AGENTS (L01)	N	1
cefaclor	ANTIBACTERIALS FOR SYSTEMIC USE (J01)	ANTIBACTERIALS FOR SYSTEMIC USE (J01)	N	1
cefadroxil	ANTIBACTERIALS FOR SYSTEMIC USE (J01)	ANTIBACTERIALS FOR SYSTEMIC USE (J01)	N	1
cefalotin	ANTIBACTERIALS FOR SYSTEMIC USE (J01)	ANTIBACTERIALS FOR SYSTEMIC USE (J01)	N	1
cefamandole	ANTIBACTERIALS FOR SYSTEMIC USE (J01)	ANTIBACTERIALS FOR SYSTEMIC USE (J01)	N	1
cefazolin	ANTIBACTERIALS FOR SYSTEMIC USE (J01)	ANTIBACTERIALS FOR SYSTEMIC USE (J01)	N	1
cefepime	ANTIBACTERIALS FOR SYSTEMIC USE (J01)	ANTIBACTERIALS FOR SYSTEMIC USE (J01)	N	1
cefixime	ANTIBACTERIALS FOR SYSTEMIC USE (J01)	ANTIBACTERIALS FOR SYSTEMIC USE (J01)	N	1
cefmetazole	ANTIBACTERIALS FOR SYSTEMIC USE (J01)	ANTIBACTERIALS FOR SYSTEMIC USE (J01)	N	1
cefoperazone	ANTIBACTERIALS FOR SYSTEMIC USE (J01)	ANTIBACTERIALS FOR SYSTEMIC USE (J01)	N	1

ceforanide	ANTIBACTERIALS FOR SYSTEMIC USE (J01)	ANTIBACTERIALS FOR SYSTEMIC USE (J01)	N	1
cefotaxime	ANTIBACTERIALS FOR SYSTEMIC USE (J01)	ANTIBACTERIALS FOR SYSTEMIC USE (J01)	N	1
cefotetan	ANTIBACTERIALS FOR SYSTEMIC USE (J01)	ANTIBACTERIALS FOR SYSTEMIC USE (J01)	N	1
cefotiam	ANTIBACTERIALS FOR SYSTEMIC USE (J01)	ANTIBACTERIALS FOR SYSTEMIC USE (J01)	N	1
cefoxitin	ANTIBACTERIALS FOR SYSTEMIC USE (J01)	ANTIBACTERIALS FOR SYSTEMIC USE (J01)	N	1
ceftazidime	ANTIBACTERIALS FOR SYSTEMIC USE (J01)	ANTIBACTERIALS FOR SYSTEMIC USE (J01)	N	1
cefuroxime	ANTIBACTERIALS FOR SYSTEMIC USE (J01)	ANTIBACTERIALS FOR SYSTEMIC USE (J01)	N	1
cetirizine	ANTIHISTAMINES FOR SYSTEMIC USE (R06)	ANTIHISTAMINES FOR SYSTEMIC USE (R06)	N	1
chlorambucil	ANTINEOPLASTIC AGENTS (L01)	ANTINEOPLASTIC AGENTS (L01)	N	1
chlorpromazine	PSYCHOLEPTICS (N05)	PSYCHOLEPTICS (N05)	N	1
chlorpropamide	DRUGS USED IN DIABETES (A10)	DRUGS USED IN DIABETES (A10)	N	1
chlorprothixene	PSYCHOLEPTICS (N05)	PSYCHOLEPTICS (N05)	N	1
chlortalidone	DIURETICS (C03)	DIURETICS (C03)	N	1
citalopram	PSYCHOANALEPTICS (N06)	PSYCHOANALEPTICS (N06)	N	1
clofibrate	LIPID MODIFYING AGENTS (C10)	LIPID MODIFYING AGENTS (C10)	N	1
clomipramine	PSYCHOANALEPTICS (N06)	PSYCHOANALEPTICS (N06)	N	1
cloxacillin	ANTIBACTERIALS FOR SYSTEMIC USE (J01)	ANTIBACTERIALS FOR SYSTEMIC USE (J01)	N	1
cyclizine	ANTIHISTAMINES FOR SYSTEMIC USE (R06)	ANTIHISTAMINES FOR SYSTEMIC USE (R06)	N	1
dacarbazine	ANTINEOPLASTIC AGENTS (L01)	ANTINEOPLASTIC AGENTS (L01)	N	1
danazol	SEX HORMONES AND MODULATORS OF THE GENITAL SYSTEM (G03)	SEX HORMONES AND MODULATORS OF THE GENITAL SYSTEM (G03)	N	1
daunorubicin	ANTINEOPLASTIC AGENTS (L01)	ANTINEOPLASTIC AGENTS (L01)	N	1
desipramine	PSYCHOANALEPTICS (N06)	PSYCHOANALEPTICS (N06)	N	1
dicloxacillin	ANTIBACTERIALS FOR SYSTEMIC USE (J01)	ANTIBACTERIALS FOR SYSTEMIC USE (J01)	N	1
diphenylpyraline	ANTIHISTAMINES FOR SYSTEMIC USE (R06)	ANTIHISTAMINES FOR SYSTEMIC USE (R06)	N	1
dirithromycin	ANTIBACTERIALS FOR SYSTEMIC USE (J01)	ANTIBACTERIALS FOR SYSTEMIC USE (J01)	N	1
dorzolamide	OPHTHALMOLOGICALS (S01)	OPHTHALMOLOGICALS (S01)	N	1
doxepin	PSYCHOANALEPTICS (N06)	PSYCHOANALEPTICS (N06)	N	1
doxorubicin	ANTINEOPLASTIC AGENTS (L01)	ANTINEOPLASTIC AGENTS (L01)	N	1
doxylamine	ANTIHISTAMINES FOR SYSTEMIC USE (R06)	ANTIHISTAMINES FOR SYSTEMIC USE (R06)	N	1
dydrogesterone	SEX HORMONES AND MODULATORS OF THE GENITAL SYSTEM (G03)	SEX HORMONES AND MODULATORS OF THE GENITAL SYSTEM (G03)	N	1
etodolac	ANTIINFLAMMATORY AND ANTIRHEUMATIC PRODUCTS (M01)	ANTIINFLAMMATORY AND ANTIRHEUMATIC PRODUCTS (M01)	N	1
felodipine	CALCIUM CHANNEL BLOCKERS (C08)	CALCIUM CHANNEL BLOCKERS (C08)	N	1
fenofibrate	LIPID MODIFYING AGENTS (C10)	LIPID MODIFYING AGENTS (C10)	N	1
fenoprofen	ANTIINFLAMMATORY AND ANTIRHEUMATIC PRODUCTS (M01)	ANTIINFLAMMATORY AND ANTIRHEUMATIC PRODUCTS (M01)	N	1
flucloxacillin	ANTIBACTERIALS FOR SYSTEMIC USE (J01)	ANTIBACTERIALS FOR SYSTEMIC USE (J01)	N	1
flufenamic_acid	ANTIINFLAMMATORY AND ANTIRHEUMATIC PRODUCTS (M01)	ANTIINFLAMMATORY AND ANTIRHEUMATIC PRODUCTS (M01)	N	1
fluoxetine	PSYCHOANALEPTICS (N06)	PSYCHOANALEPTICS (N06)	N	1

fluphenazine	PSYCHOLEPTICS (N05)	PSYCHOLEPTICS (N05)	N	1
fluspirilene	PSYCHOLEPTICS (N05)	PSYCHOLEPTICS (N05)	N	1
fluvastatin	LIPID MODIFYING AGENTS (C10)	LIPID MODIFYING AGENTS (C10)	N	1
fluvoxamine	PSYCHOANALEPTICS (N06)	PSYCHOANALEPTICS (N06)	N	1
furosemide	DIURETICS (C03)	DIURETICS (C03)	N	1
gliclazide	DRUGS USED IN DIABETES (A10)	DRUGS USED IN DIABETES (A10)	N	1
glimepiride	DRUGS USED IN DIABETES (A10)	DRUGS USED IN DIABETES (A10)	N	1
glipizide	DRUGS USED IN DIABETES (A10)	DRUGS USED IN DIABETES (A10)	N	1
gliquidone	DRUGS USED IN DIABETES (A10)	DRUGS USED IN DIABETES (A10)	N	1
haloperidol	PSYCHOLEPTICS (N05)	PSYCHOLEPTICS (N05)	N	1
hydrochlorothiazide	DIURETICS (C03)	DIURETICS (C03)	N	1
hydroflumethiazide	DIURETICS (C03)	DIURETICS (C03)	N	1
ifosfamide	ANTINEOPLASTIC AGENTS (L01)	ANTINEOPLASTIC AGENTS (L01)	N	1
imipramine	PSYCHOANALEPTICS (N06)	PSYCHOANALEPTICS (N06)	N	1
isradipine	CALCIUM CHANNEL BLOCKERS (C08)	CALCIUM CHANNEL BLOCKERS (C08)	N	1
josamycin	ANTIBACTERIALS FOR SYSTEMIC USE (J01)	ANTIBACTERIALS FOR SYSTEMIC USE (J01)	N	1
latamoxef	ANTIBACTERIALS FOR SYSTEMIC USE (J01)	ANTIBACTERIALS FOR SYSTEMIC USE (J01)	N	1
lincomycin	ANTIBACTERIALS FOR SYSTEMIC USE (J01)	ANTIBACTERIALS FOR SYSTEMIC USE (J01)	N	1
lomustine	ANTINEOPLASTIC AGENTS (L01)	ANTINEOPLASTIC AGENTS (L01)	N	1
loracarbef	ANTIBACTERIALS FOR SYSTEMIC USE (J01)	ANTIBACTERIALS FOR SYSTEMIC USE (J01)	N	1
lovastatin	LIPID MODIFYING AGENTS (C10)	LIPID MODIFYING AGENTS (C10)	N	1
loxapine	PSYCHOLEPTICS (N05)	PSYCHOLEPTICS (N05)	N	1
maprotiline	PSYCHOANALEPTICS (N06)	PSYCHOANALEPTICS (N06)	N	1
mefenamic_acid	ANTIINFLAMMATORY AND ANTIRHEUMATIC PRODUCTS (M01)	ANTIINFLAMMATORY AND ANTIRHEUMATIC PRODUCTS (M01)	N	1
meropenem	ANTIBACTERIALS FOR SYSTEMIC USE (J01)	ANTIBACTERIALS FOR SYSTEMIC USE (J01)	N	1
mesoridazine	PSYCHOLEPTICS (N05)	PSYCHOLEPTICS (N05)	N	1
metaraminol	CARDIAC THERAPY (C01)	CARDIAC THERAPY (C01)	N	1
methazolamide	OPHTHALMOLOGICALS (S01)	OPHTHALMOLOGICALS (S01)	N	1
methoxamine	CARDIAC THERAPY (C01)	CARDIAC THERAPY (C01)	N	1
metolazone	DIURETICS (C03)	DIURETICS (C03)	N	1
metoprolol	BETA BLOCKING AGENTS (C07)	BETA BLOCKING AGENTS (C07)	N	1
mianserin	PSYCHOANALEPTICS (N06)	PSYCHOANALEPTICS (N06)	N	1
minaprine	PSYCHOANALEPTICS (N06)	PSYCHOANALEPTICS (N06)	N	1
mitoxantrone	ANTINEOPLASTIC AGENTS (L01)	ANTINEOPLASTIC AGENTS (L01)	N	1
molindone	PSYCHOLEPTICS (N05)	PSYCHOLEPTICS (N05)	N	1
nabumetone	ANTIINFLAMMATORY AND ANTIRHEUMATIC PRODUCTS (M01)	ANTIINFLAMMATORY AND ANTIRHEUMATIC PRODUCTS (M01)	N	1
nadolol	BETA BLOCKING AGENTS (C07)	BETA BLOCKING AGENTS (C07)	N	1

nicardipine	CALCIUM CHANNEL BLOCKERS (C08)	CALCIUM CHANNEL BLOCKERS (C08)	N	1
nifedipine	CALCIUM CHANNEL BLOCKERS (C08)	CALCIUM CHANNEL BLOCKERS (C08)	N	1
nimodipine	CALCIUM CHANNEL BLOCKERS (C08)	CALCIUM CHANNEL BLOCKERS (C08)	N	1
nitrendipine	CALCIUM CHANNEL BLOCKERS (C08)	CALCIUM CHANNEL BLOCKERS (C08)	N	1
nitrofurantoin	ANTIBACTERIALS FOR SYSTEMIC USE (J01)	ANTIBACTERIALS FOR SYSTEMIC USE (J01)	N	1
nortriptyline	PSYCHOANALEPTICS (N06)	PSYCHOANALEPTICS (N06)	N	1
oxaprozin	ANTIINFLAMMATORY AND ANTIRHEUMATIC PRODUCTS (M01)	ANTIINFLAMMATORY AND ANTIRHEUMATIC PRODUCTS (M01)	N	1
oxprenolol	BETA BLOCKING AGENTS (C07)	BETA BLOCKING AGENTS (C07)	N	1
paroxetine	PSYCHOANALEPTICS (N06)	PSYCHOANALEPTICS (N06)	N	1
penbutolol	BETA BLOCKING AGENTS (C07)	BETA BLOCKING AGENTS (C07)	N	1
perphenazine	PSYCHOLEPTICS (N05)	PSYCHOLEPTICS (N05)	N	1
pheniramine	ANTIHISTAMINES FOR SYSTEMIC USE (R06)	ANTIHISTAMINES FOR SYSTEMIC USE (R06)	N	1
pimozide	PSYCHOLEPTICS (N05)	PSYCHOLEPTICS (N05)	N	1
pindolol	BETA BLOCKING AGENTS (C07)	BETA BLOCKING AGENTS (C07)	N	1
piperacillin	ANTIBACTERIALS FOR SYSTEMIC USE (J01)	ANTIBACTERIALS FOR SYSTEMIC USE (J01)	N	1
piretanide	DIURETICS (C03)	DIURETICS (C03)	N	1
pivampicillin	ANTIBACTERIALS FOR SYSTEMIC USE (J01)	ANTIBACTERIALS FOR SYSTEMIC USE (J01)	N	1
pivmecillinam	ANTIBACTERIALS FOR SYSTEMIC USE (J01)	ANTIBACTERIALS FOR SYSTEMIC USE (J01)	N	1
practolol	BETA BLOCKING AGENTS (C07)	BETA BLOCKING AGENTS (C07)	N	1
procarbazine	ANTINEOPLASTIC AGENTS (L01)	ANTINEOPLASTIC AGENTS (L01)	N	1
prochlorperazine	PSYCHOLEPTICS (N05)	PSYCHOLEPTICS (N05)	N	1
progesterone	SEX HORMONES AND MODULATORS OF THE GENITAL SYSTEM (G03)	SEX HORMONES AND MODULATORS OF THE GENITAL SYSTEM (G03)	N	1
propranolol	BETA BLOCKING AGENTS (C07)	BETA BLOCKING AGENTS (C07)	N	1
protriptyline	PSYCHOANALEPTICS (N06)	PSYCHOANALEPTICS (N06)	N	1
quinethazone	DIURETICS (C03)	DIURETICS (C03)	N	1
repaglinide	DRUGS USED IN DIABETES (A10)	DRUGS USED IN DIABETES (A10)	N	1
rofecoxib	ANTIINFLAMMATORY AND ANTIRHEUMATIC PRODUCTS (M01)	ANTIINFLAMMATORY AND ANTIRHEUMATIC PRODUCTS (M01)	N	1
roxithromycin	ANTIBACTERIALS FOR SYSTEMIC USE (J01)	ANTIBACTERIALS FOR SYSTEMIC USE (J01)	N	1
simvastatin	LIPID MODIFYING AGENTS (C10)	LIPID MODIFYING AGENTS (C10)	N	1
spectinomycin	ANTIBACTERIALS FOR SYSTEMIC USE (J01)	ANTIBACTERIALS FOR SYSTEMIC USE (J01)	N	1
sulfadimethoxine	ANTIBACTERIALS FOR SYSTEMIC USE (J01)	ANTIBACTERIALS FOR SYSTEMIC USE (J01)	N	1
sulfapyridine	ANTIBACTERIALS FOR SYSTEMIC USE (J01)	ANTIBACTERIALS FOR SYSTEMIC USE (J01)	N	1
sulindac	ANTIINFLAMMATORY AND ANTIRHEUMATIC PRODUCTS (M01)	ANTIINFLAMMATORY AND ANTIRHEUMATIC PRODUCTS (M01)	N	1
sulpiride	PSYCHOLEPTICS (N05)	PSYCHOLEPTICS (N05)	N	1
suprofen	ANTIINFLAMMATORY AND ANTIRHEUMATIC PRODUCTS (M01)	ANTIINFLAMMATORY AND ANTIRHEUMATIC PRODUCTS (M01)	N	1
tenoxicam	ANTIINFLAMMATORY AND ANTIRHEUMATIC PRODUCTS (M01)	ANTIINFLAMMATORY AND ANTIRHEUMATIC PRODUCTS (M01)	N	1
terfenadine	ANTIHISTAMINES FOR SYSTEMIC USE (R06)	ANTIHISTAMINES FOR SYSTEMIC USE (R06)	N	1

thioproperazine	PSYCHOLEPTICS (N05)	PSYCHOLEPTICS (N05)	N	1
thioridazine	PSYCHOLEPTICS (N05)	PSYCHOLEPTICS (N05)	N	1
tiaprofenic_acid	ANTIINFLAMMATORY AND ANTIRHEUMATIC PRODUCTS (M01)	ANTIINFLAMMATORY AND ANTIRHEUMATIC PRODUCTS (M01)	N	1
ticarcillin	ANTIBACTERIALS FOR SYSTEMIC USE (J01)	ANTIBACTERIALS FOR SYSTEMIC USE (J01)	N	1
tioguanine	ANTINEOPLASTIC AGENTS (L01)	ANTINEOPLASTIC AGENTS (L01)	N	1
torasemide	DIURETICS (C03)	DIURETICS (C03)	N	1
trazodone	PSYCHOANALEPTICS (N06)	PSYCHOANALEPTICS (N06)	N	1
trichlormethiazide	DIURETICS (C03)	DIURETICS (C03)	N	1
trifluoperazine	PSYCHOLEPTICS (N05)	PSYCHOLEPTICS (N05)	N	1
triflupromazine	PSYCHOLEPTICS (N05)	PSYCHOLEPTICS (N05)	N	1
trimipramine	PSYCHOANALEPTICS (N06)	PSYCHOANALEPTICS (N06)	N	1
triprolidine	ANTIHISTAMINES FOR SYSTEMIC USE (R06)	ANTIHISTAMINES FOR SYSTEMIC USE (R06)	N	1
troleandomycin	ANTIBACTERIALS FOR SYSTEMIC USE (J01)	ANTIBACTERIALS FOR SYSTEMIC USE (J01)	N	1
zuclopenthixol	PSYCHOLEPTICS (N05)	PSYCHOLEPTICS (N05)	N	1
pergolide	ANTI-PARKINSON DRUGS (N04)	PSYCHOLEPTICS (N05)	Y	0.99
bendroflumethiazide	DIURETICS (C03)	DIURETICS (C03)	N	0.99
bezafibrate	LIPID MODIFYING AGENTS (C10)	LIPID MODIFYING AGENTS (C10)	N	0.99
buspirone	PSYCHOLEPTICS (N05)	PSYCHOLEPTICS (N05)	N	0.99
clomifene	SEX HORMONES AND MODULATORS OF THE GENITAL SYSTEM (G03)	SEX HORMONES AND MODULATORS OF THE GENITAL SYSTEM (G03)	N	0.99
etoposide	ANTINEOPLASTIC AGENTS (L01)	ANTINEOPLASTIC AGENTS (L01)	N	0.99
gemfibrozil	LIPID MODIFYING AGENTS (C10)	LIPID MODIFYING AGENTS (C10)	N	0.99
mexiletine	CARDIAC THERAPY (C01)	CARDIAC THERAPY (C01)	N	0.99
midodrine	CARDIAC THERAPY (C01)	CARDIAC THERAPY (C01)	N	0.99
propafenone	CARDIAC THERAPY (C01)	CARDIAC THERAPY (C01)	N	0.99
quinidine	CARDIAC THERAPY (C01)	CARDIAC THERAPY (C01)	N	0.99
remoxipride	PSYCHOLEPTICS (N05)	PSYCHOLEPTICS (N05)	N	0.99
risperidone	PSYCHOLEPTICS (N05)	PSYCHOLEPTICS (N05)	N	0.99
sulfadiazine	ANTIBACTERIALS FOR SYSTEMIC USE (J01)	ANTIBACTERIALS FOR SYSTEMIC USE (J01)	N	0.99
amiodarone	CARDIAC THERAPY (C01)	BETA BLOCKING AGENTS (C07)	Y	0.98
nalidixic_acid	ANTIBACTERIALS FOR SYSTEMIC USE (J01)	ANTINEOPLASTIC AGENTS (L01)	Y	0.98
pargyline	ANTIHYPERTENSIVES (C02)	PSYCHOANALEPTICS (N06)	Y	0.98
selegiline	ANTI-PARKINSON DRUGS (N04)	PSYCHOANALEPTICS (N06)	Y	0.98
trifluridine	OPHTHALMOLOGICALS (S01)	ANTINEOPLASTIC AGENTS (L01)	Y	0.98
trimethoprim	ANTIBACTERIALS FOR SYSTEMIC USE (J01)	ANTIPROTOZOALS (P01)	Y	0.98
acetohexamide	DRUGS USED IN DIABETES (A10)	DRUGS USED IN DIABETES (A10)	N	0.98
amoxapine	PSYCHOANALEPTICS (N06)	PSYCHOANALEPTICS (N06)	N	0.98
mephentermine	CARDIAC THERAPY (C01)	CARDIAC THERAPY (C01)	N	0.98

promazine	PSYCHOLEPTICS (N05)	PSYCHOLEPTICS (N05)	N	0.98
raloxifene	SEX HORMONES AND MODULATORS OF THE GENITAL SYSTEM (G03)	SEX HORMONES AND MODULATORS OF THE GENITAL SYSTEM (G03)	N	0.98
tolazamide	DRUGS USED IN DIABETES (A10)	DRUGS USED IN DIABETES (A10)	N	0.98
praziquantel	ANTHELMINTICS (P02)	ANTIEPILEPTICS (N03)	Y	0.97
vinblastine	ANTINEOPLASTIC AGENTS (L01)	ANTIBACTERIALS FOR SYSTEMIC USE (J01)	Y	0.97
pioglitazone	DRUGS USED IN DIABETES (A10)	DRUGS USED IN DIABETES (A10)	N	0.97
estradiol	SEX HORMONES AND MODULATORS OF THE GENITAL SYSTEM (G03)	SEX HORMONES AND MODULATORS OF THE GENITAL SYSTEM (G03)	N	0.96
flecainide	CARDIAC THERAPY (C01)	CARDIAC THERAPY (C01)	N	0.96
sotalol	BETA BLOCKING AGENTS (C07)	BETA BLOCKING AGENTS (C07)	N	0.96
altretamine	ANTINEOPLASTIC AGENTS (L01)	ANTINEOPLASTIC AGENTS (L01)	N	0.95
ajmaline	CARDIAC THERAPY (C01)	CARDIAC THERAPY (C01)	N	0.94
brompheniramine	ANTIHISTAMINES FOR SYSTEMIC USE (R06)	ANTIHISTAMINES FOR SYSTEMIC USE (R06)	N	0.94
chloroquine	ANTIPROTOZOALS (P01)	ANTIPROTOZOALS (P01)	N	0.94
trihexyphenidyl	ANTI-PARKINSON DRUGS (N04)	ANTI-PARKINSON DRUGS (N04)	N	0.94
nomifensine	PSYCHOANALEPTICS (N06)	PSYCHOANALEPTICS (N06)	N	0.93
primidone	ANTIEPILEPTICS (N03)	ANTIEPILEPTICS (N03)	N	0.93
proxymetacaine	OPHTHALMOLOGICALS (S01)	ANTIBACTERIALS FOR SYSTEMIC USE (J01)	Y	0.92
mercaptopurine	ANTINEOPLASTIC AGENTS (L01)	ANTINEOPLASTIC AGENTS (L01)	N	0.92
tacrine	PSYCHOANALEPTICS (N06)	PSYCHOANALEPTICS (N06)	N	0.92
biperiden	ANTI-PARKINSON DRUGS (N04)	ANTIHYPERTENSIVES (C02)	Y	0.91
amrinone	CARDIAC THERAPY (C01)	CARDIAC THERAPY (C01)	N	0.91
lymecycline	ANTIBACTERIALS FOR SYSTEMIC USE (J01)	ANTIBACTERIALS FOR SYSTEMIC USE (J01)	N	0.91
mifepristone	SEX HORMONES AND MODULATORS OF THE GENITAL SYSTEM (G03)	SEX HORMONES AND MODULATORS OF THE GENITAL SYSTEM (G03)	N	0.91
procyclidine	ANTI-PARKINSON DRUGS (N04)	ANTI-PARKINSON DRUGS (N04)	N	0.91
proguanil	ANTIPROTOZOALS (P01)	ANTIPROTOZOALS (P01)	N	0.91
vigabatrin	ANTIEPILEPTICS (N03)	ANTIEPILEPTICS (N03)	N	0.91
dopamine	CARDIAC THERAPY (C01)	ANTI-PARKINSON DRUGS (N04)	Y	0.9
gabapentin	ANTIEPILEPTICS (N03)	CALCIUM CHANNEL BLOCKERS (C08)	Y	0.9
mecamylamine	ANTIHYPERTENSIVES (C02)	ANTI-PARKINSON DRUGS (N04)	Y	0.9
memantine	PSYCHOANALEPTICS (N06)	ANTI-PARKINSON DRUGS (N04)	Y	0.9
pyrimethamine	ANTIPROTOZOALS (P01)	ANTIBACTERIALS FOR SYSTEMIC USE (J01)	Y	0.9
albendazole	ANTHELMINTICS (P02)	ANTHELMINTICS (P02)	N	0.9
cyproterone	SEX HORMONES AND MODULATORS OF THE GENITAL SYSTEM (G03)	SEX HORMONES AND MODULATORS OF THE GENITAL SYSTEM (G03)	N	0.9
enoxacin	ANTIBACTERIALS FOR SYSTEMIC USE (J01)	ANTIBACTERIALS FOR SYSTEMIC USE (J01)	N	0.9
mebendazole	ANTHELMINTICS (P02)	ANTHELMINTICS (P02)	N	0.9
milrinone	CARDIAC THERAPY (C01)	CARDIAC THERAPY (C01)	N	0.9
phenformin	DRUGS USED IN DIABETES (A10)	DRUGS USED IN DIABETES (A10)	N	0.9

prazosin	ANTIHYPERTENSIVES (C02)	ANTIHYPERTENSIVES (C02)	N	0.9
rolitetracycline	ANTIBACTERIALS FOR SYSTEMIC USE (J01)	ANTIBACTERIALS FOR SYSTEMIC USE (J01)	N	0.9
rosiglitazone	DRUGS USED IN DIABETES (A10)	DRUGS USED IN DIABETES (A10)	N	0.9
testosterone	SEX HORMONES AND MODULATORS OF THE GENITAL SYSTEM (G03)	SEX HORMONES AND MODULATORS OF THE GENITAL SYSTEM (G03)	N	0.9
trimethadione	ANTIEPILEPTICS (N03)	ANTIEPILEPTICS (N03)	N	0.9
azacitidine	ANTINEOPLASTIC AGENTS (L01)	OPHTHALMOLOGICALS (S01)	Y	0.89
cyclopentolate	OPHTHALMOLOGICALS (S01)	ANTI-PARKINSON DRUGS (N04)	Y	0.89
hydralazine	ANTIHYPERTENSIVES (C02)	PSYCHOANALEPTICS (N06)	Y	0.89
amiloride	DIURETICS (C03)	DIURETICS (C03)	N	0.89
amodiaquine	ANTIPROTOZOALS (P01)	ANTIPROTOZOALS (P01)	N	0.89
bupropion	PSYCHOANALEPTICS (N06)	PSYCHOANALEPTICS (N06)	N	0.89
doxazosin	ANTIHYPERTENSIVES (C02)	ANTIHYPERTENSIVES (C02)	N	0.89
ethosuximide	ANTIEPILEPTICS (N03)	ANTIEPILEPTICS (N03)	N	0.89
irinotecan	ANTINEOPLASTIC AGENTS (L01)	ANTINEOPLASTIC AGENTS (L01)	N	0.89
metformin	DRUGS USED IN DIABETES (A10)	DRUGS USED IN DIABETES (A10)	N	0.89
primaquine	ANTIPROTOZOALS (P01)	ANTIPROTOZOALS (P01)	N	0.89
triamterene	DIURETICS (C03)	DIURETICS (C03)	N	0.89
melatonin	PSYCHOLEPTICS (N05)	ANTIPROTOZOALS (P01)	Y	0.88
tranylcypromine	PSYCHOANALEPTICS (N06)	ANTI-PARKINSON DRUGS (N04)	Y	0.88
valproic_acid	ANTIEPILEPTICS (N03)	ANTIEPILEPTICS (N03)	N	0.88
verteporfin	OPHTHALMOLOGICALS (S01)	ANTINEOPLASTIC AGENTS (L01)	Y	0.87
metixene	ANTI-PARKINSON DRUGS (N04)	PSYCHOLEPTICS (N05)	Y	0.86
sulfamethoxazole	ANTIBACTERIALS FOR SYSTEMIC USE (J01)	OPHTHALMOLOGICALS (S01)	Y	0.86
tropicamide	OPHTHALMOLOGICALS (S01)	ANTI-PARKINSON DRUGS (N04)	Y	0.84
diloxanide	ANTIPROTOZOALS (P01)	ANTIBACTERIALS FOR SYSTEMIC USE (J01)	Y	0.83
levodopa	ANTI-PARKINSON DRUGS (N04)	CARDIAC THERAPY (C01)	Y	0.81
cinoxacin	ANTIBACTERIALS FOR SYSTEMIC USE (J01)	ANTIBACTERIALS FOR SYSTEMIC USE (J01)	N	0.8
galantamine	PSYCHOANALEPTICS (N06)	ANTIHYPERTENSIVES (C02)	Y	0.79
indapamide	DIURETICS (C03)	DIURETICS (C03)	N	0.79
tocainide	CARDIAC THERAPY (C01)	CARDIAC THERAPY (C01)	N	0.79
diethylcarbamazine	ANTHELMINTICS (P02)	ANTINEOPLASTIC AGENTS (L01)	Y	0.78
levamisole	ANTHELMINTICS (P02)	DRUGS USED IN DIABETES (A10)	Y	0.78
vorinostat	ANTINEOPLASTIC AGENTS (L01)	LIPID MODIFYING AGENTS (C10)	Y	0.77
ethotoin	ANTIEPILEPTICS (N03)	CARDIAC THERAPY (C01)	Y	0.75
amantadine	ANTI-PARKINSON DRUGS (N04)	PSYCHOANALEPTICS (N06)	Y	0.73
atovaquone	ANTIPROTOZOALS (P01)	SEX HORMONES AND MODULATORS OF THE GENITAL SYSTEM (G03)	Y	0.7
procainamide	CARDIAC THERAPY (C01)	ANTIEPILEPTICS (N03)	Y	0.7

labetalol	BETA BLOCKING AGENTS (C07)	CARDIAC THERAPY (C01)	Y	0.69
guanfacine	ANTIHYPERTENSIVES (C02)	ANTIEPILEPTICS (N03)	Y	0.68
imatinib	ANTINEOPLASTIC AGENTS (L01)	DIURETICS (C03)	Y	0.68
reserpine	ANTIHYPERTENSIVES (C02)	ANTIHYPERTENSIVES (C02)	N	0.67
medrysone	OPHTHALMOLOGICALS (S01)	SEX HORMONES AND MODULATORS OF THE GENITAL SYSTEM (G03)	Y	0.66
probucol	LIPID MODIFYING AGENTS (C10)	ANTIBACTERIALS FOR SYSTEMIC USE (J01)	Y	0.66
halofantrine	ANTIPROTOZOALS (P01)	ANTIHISTAMINES FOR SYSTEMIC USE (R06)	Y	0.64
mephenytoin	ANTIEPILEPTICS (N03)	ANTIEPILEPTICS (N03)	N	0.64
streptozocin	ANTINEOPLASTIC AGENTS (L01)	ANTIBACTERIALS FOR SYSTEMIC USE (J01)	Y	0.62
clozapine	PSYCHOLEPTICS (N05)	PSYCHOLEPTICS (N05)	N	0.61
perhexiline	CALCIUM CHANNEL BLOCKERS (C08)	ANTI-PARKINSON DRUGS (N04)	Y	0.6
phensuximide	ANTIEPILEPTICS (N03)	ANTIEPILEPTICS (N03)	N	0.6
cyproheptadine	ANTIHISTAMINES FOR SYSTEMIC USE (R06)	PSYCHOLEPTICS (N05)	Y	0.59
disopyramide	CARDIAC THERAPY (C01)	ANTIHISTAMINES FOR SYSTEMIC USE (R06)	Y	0.58
verapamil	CALCIUM CHANNEL BLOCKERS (C08)	CALCIUM CHANNEL BLOCKERS (C08)	N	0.58
bepridil	CALCIUM CHANNEL BLOCKERS (C08)	CALCIUM CHANNEL BLOCKERS (C08)	N	0.56
diltiazem	CALCIUM CHANNEL BLOCKERS (C08)	ANTIINFLAMMATORY AND ANTIRHEUMATIC PRODUCTS (M01)	Y	0.52
isocarboxazid	PSYCHOANALEPTICS (N06)	ANTIBACTERIALS FOR SYSTEMIC USE (J01)	Y	0.52
rescinnamine	ANTIHYPERTENSIVES (C02)	ANTIHYPERTENSIVES (C02)	N	0.52
digoxin	CARDIAC THERAPY (C01)	DIURETICS (C03)	Y	0.51
mefloquine	ANTIPROTOZOALS (P01)	ANTHELMINTICS (P02)	Y	0.48
digoxin	CARDIAC THERAPY (C01)	ANTIBACTERIALS FOR SYSTEMIC USE (J01)	Y	0.47
rescinnamine	ANTIHYPERTENSIVES (C02)	ANTINEOPLASTIC AGENTS (L01)	Y	0.47
isocarboxazid	PSYCHOANALEPTICS (N06)	ANTIHYPERTENSIVES (C02)	Y	0.45
phenelzine	PSYCHOANALEPTICS (N06)	ANTIEPILEPTICS (N03)	Y	0.42
verapamil	CALCIUM CHANNEL BLOCKERS (C08)	CARDIAC THERAPY (C01)	Y	0.42
bepridil	CALCIUM CHANNEL BLOCKERS (C08)	CARDIAC THERAPY (C01)	Y	0.41
cyproheptadine	ANTIHISTAMINES FOR SYSTEMIC USE (R06)	PSYCHOANALEPTICS (N06)	Y	0.41
phensuximide	ANTIEPILEPTICS (N03)	ANTIBACTERIALS FOR SYSTEMIC USE (J01)	Y	0.4
clozapine	PSYCHOLEPTICS (N05)	PSYCHOANALEPTICS (N06)	Y	0.39
streptozocin	ANTINEOPLASTIC AGENTS (L01)	ANTINEOPLASTIC AGENTS (L01)	N	0.38
mephenytoin	ANTIEPILEPTICS (N03)	CARDIAC THERAPY (C01)	Y	0.36
disopyramide	CARDIAC THERAPY (C01)	ANTIEPILEPTICS (N03)	Y	0.35
medrysone	OPHTHALMOLOGICALS (S01)	ANTIBACTERIALS FOR SYSTEMIC USE (J01)	Y	0.34
probucol	LIPID MODIFYING AGENTS (C10)	CARDIAC THERAPY (C01)	Y	0.34
halofantrine	ANTIPROTOZOALS (P01)	CARDIAC THERAPY (C01)	Y	0.33
diltiazem	CALCIUM CHANNEL BLOCKERS (C08)	ANTIBACTERIALS FOR SYSTEMIC USE (J01)	Y	0.32

perhexiline	CALCIUM CHANNEL BLOCKERS (C08)	ANTIBACTERIALS FOR SYSTEMIC USE (J01)	Y	0.32
phenelzine	PSYCHOANALEPTICS (N06)	PSYCHOANALEPTICS (N06)	N	0.32
reserpine	ANTIHYPERTENSIVES (C02)	ANTIBACTERIALS FOR SYSTEMIC USE (J01)	Y	0.31
labetalol	BETA BLOCKING AGENTS (C07)	BETA BLOCKING AGENTS (C07)	N	0.31
guanfacine	ANTIHYPERTENSIVES (C02)	PSYCHOANALEPTICS (N06)	Y	0.29
imatinib	ANTINEOPLASTIC AGENTS (L01)	ANTIBACTERIALS FOR SYSTEMIC USE (J01)	Y	0.28
amantadine	ANTI-PARKINSON DRUGS (N04)	PSYCHOLEPTICS (N05)	Y	0.27
paclitaxel	ANTINEOPLASTIC AGENTS (L01)	ANTIBACTERIALS FOR SYSTEMIC USE (J01)	Y	0.27
ethotoin	ANTIEPILEPTICS (N03)	ANTIEPILEPTICS (N03)	N	0.25
mefloquine	ANTIPROTOZOALS (P01)	ANTI-PARKINSON DRUGS (N04)	Y	0.24
diethylcarbamazine	ANTHELMINTICS (P02)	ANTIINFLAMMATORY AND ANTIRHEUMATIC PRODUCTS (M01)	Y	0.22
paclitaxel	ANTINEOPLASTIC AGENTS (L01)	DIURETICS (C03)	Y	0.22
phenelzine	PSYCHOANALEPTICS (N06)	CARDIAC THERAPY (C01)	Y	0.22
tocainide	CARDIAC THERAPY (C01)	ANTIEPILEPTICS (N03)	Y	0.21
cinoxacin	ANTIBACTERIALS FOR SYSTEMIC USE (J01)	ANTINEOPLASTIC AGENTS (L01)	Y	0.2
levodopa	ANTI-PARKINSON DRUGS (N04)	PSYCHOLEPTICS (N05)	Y	0.19
procainamide	CARDIAC THERAPY (C01)	CARDIAC THERAPY (C01)	N	0.19
vorinostat	ANTINEOPLASTIC AGENTS (L01)	ANTINEOPLASTIC AGENTS (L01)	N	0.17
indapamide	DIURETICS (C03)	ANTIBACTERIALS FOR SYSTEMIC USE (J01)	Y	0.15
tropicamide	OPHTHALMOLOGICALS (S01)	ANTINEOPLASTIC AGENTS (L01)	Y	0.15
sulfamethoxazole	ANTIBACTERIALS FOR SYSTEMIC USE (J01)	ANTIBACTERIALS FOR SYSTEMIC USE (J01)	N	0.14
diloxanide	ANTIPROTOZOALS (P01)	DIURETICS (C03)	Y	0.13
levamisole	ANTHELMINTICS (P02)	ANTIPROTOZOALS (P01)	Y	0.13
paclitaxel	ANTINEOPLASTIC AGENTS (L01)	CALCIUM CHANNEL BLOCKERS (C08)	Y	0.13
paclitaxel	ANTINEOPLASTIC AGENTS (L01)	ANTIEPILEPTICS (N03)	Y	0.13
atovaquone	ANTIPROTOZOALS (P01)	CARDIAC THERAPY (C01)	Y	0.12
valproic_acid	ANTIEPILEPTICS (N03)	ANTINEOPLASTIC AGENTS (L01)	Y	0.12
paclitaxel	ANTINEOPLASTIC AGENTS (L01)	ANTINEOPLASTIC AGENTS (L01)	N	0.12
amiloride	DIURETICS (C03)	ANTIBACTERIALS FOR SYSTEMIC USE (J01)	Y	0.11
atovaquone	ANTIPROTOZOALS (P01)	DIURETICS (C03)	Y	0.11
mefloquine	ANTIPROTOZOALS (P01)	PSYCHOLEPTICS (N05)	Y	0.11
azacitidine	ANTINEOPLASTIC AGENTS (L01)	ANTINEOPLASTIC AGENTS (L01)	N	0.11
bupropion	PSYCHOANALEPTICS (N06)	CARDIAC THERAPY (C01)	Y	0.1
procainamide	CARDIAC THERAPY (C01)	ANTINEOPLASTIC AGENTS (L01)	Y	0.1
tranylcypromine	PSYCHOANALEPTICS (N06)	PSYCHOANALEPTICS (N06)	N	0.1
dopamine	CARDIAC THERAPY (C01)	PSYCHOLEPTICS (N05)	Y	0.098
galantamine	PSYCHOANALEPTICS (N06)	ANTINEOPLASTIC AGENTS (L01)	Y	0.098

enoxacin	ANTIBACTERIALS FOR SYSTEMIC USE (J01)	ANTINEOPLASTIC AGENTS (L01)	Y	0.095
rosiglitazone	DRUGS USED IN DIABETES (A10)	ANTIBACTERIALS FOR SYSTEMIC USE (J01)	Y	0.095
cyproterone	SEX HORMONES AND MODULATORS OF THE GENITAL SYSTEM (G03)	DIURETICS (C03)	Y	0.093
proguanil	ANTIPROTOZOALS (P01)	ANTIBACTERIALS FOR SYSTEMIC USE (J01)	Y	0.093
pyrimethamine	ANTIPROTOZOALS (P01)	ANTIPROTOZOALS (P01)	N	0.093
memantine	PSYCHOANALEPTICS (N06)	ANTIBACTERIALS FOR SYSTEMIC USE (J01)	Y	0.092
albendazole	ANTHELMINTICS (P02)	ANTINEOPLASTIC AGENTS (L01)	Y	0.089
verteporfin	OPHTHALMOLOGICALS (S01)	ANTIHYPERTENSIVES (C02)	Y	0.088
paclitaxel	ANTINEOPLASTIC AGENTS (L01)	BETA BLOCKING AGENTS (C07)	Y	0.087
lymecycline	ANTIBACTERIALS FOR SYSTEMIC USE (J01)	ANTINEOPLASTIC AGENTS (L01)	Y	0.086
primaquine	ANTIPROTOZOALS (P01)	PSYCHOLEPTICS (N05)	Y	0.086
mifepristone	SEX HORMONES AND MODULATORS OF THE GENITAL SYSTEM (G03)	OPHTHALMOLOGICALS (S01)	Y	0.085
metixene	ANTI-PARKINSON DRUGS (N04)	ANTI-PARKINSON DRUGS (N04)	N	0.085
amrinone	CARDIAC THERAPY (C01)	ANTIPROTOZOALS (P01)	Y	0.084
mebendazole	ANTHELMINTICS (P02)	ANTINEOPLASTIC AGENTS (L01)	Y	0.083
testosterone	SEX HORMONES AND MODULATORS OF THE GENITAL SYSTEM (G03)	DIURETICS (C03)	Y	0.081
vigabatrin	ANTIEPILEPTICS (N03)	ANTIBACTERIALS FOR SYSTEMIC USE (J01)	Y	0.08
diltiazem	CALCIUM CHANNEL BLOCKERS (C08)	PSYCHOLEPTICS (N05)	Y	0.078
mecamylamine	ANTIHYPERTENSIVES (C02)	CALCIUM CHANNEL BLOCKERS (C08)	Y	0.078
mefloquine	ANTIPROTOZOALS (P01)	CARDIAC THERAPY (C01)	Y	0.078
phenformin	DRUGS USED IN DIABETES (A10)	ANTIEPILEPTICS (N03)	Y	0.077
mefloquine	ANTIPROTOZOALS (P01)	DIURETICS (C03)	Y	0.076
disopyramide	CARDIAC THERAPY (C01)	CARDIAC THERAPY (C01)	N	0.074
primidone	ANTIEPILEPTICS (N03)	ANTIBACTERIALS FOR SYSTEMIC USE (J01)	Y	0.072
hydralazine	ANTIHYPERTENSIVES (C02)	ANTINEOPLASTIC AGENTS (L01)	Y	0.07
melatonin	PSYCHOLEPTICS (N05)	ANTIBACTERIALS FOR SYSTEMIC USE (J01)	Y	0.069
biperiden	ANTI-PARKINSON DRUGS (N04)	ANTI-PARKINSON DRUGS (N04)	N	0.069
milrinone	CARDIAC THERAPY (C01)	ANTIPROTOZOALS (P01)	Y	0.068
nomifensine	PSYCHOANALEPTICS (N06)	ANTIHISTAMINES FOR SYSTEMIC USE (R06)	Y	0.068
gabapentin	ANTIEPILEPTICS (N03)	CARDIAC THERAPY (C01)	Y	0.067
indapamide	DIURETICS (C03)	PSYCHOANALEPTICS (N06)	Y	0.067
irinotecan	ANTINEOPLASTIC AGENTS (L01)	OPHTHALMOLOGICALS (S01)	Y	0.065
triamterene	DIURETICS (C03)	ANTIBACTERIALS FOR SYSTEMIC USE (J01)	Y	0.065
ajmaline	CARDIAC THERAPY (C01)	ANTIEPILEPTICS (N03)	Y	0.062
chloroquine	ANTIPROTOZOALS (P01)	CARDIAC THERAPY (C01)	Y	0.06
procyclidine	ANTI-PARKINSON DRUGS (N04)	ANTIHISTAMINES FOR SYSTEMIC USE (R06)	Y	0.059
proxymetacaine	OPHTHALMOLOGICALS (S01)	ANTINEOPLASTIC AGENTS (L01)	Y	0.059

doxazosin	ANTIHYPERTENSIVES (C02)	PSYCHOANALEPTICS (N06)	Y	0.058
metixene	ANTI-PARKINSON DRUGS (N04)	PSYCHOANALEPTICS (N06)	Y	0.058
rolitetracycline	ANTIBACTERIALS FOR SYSTEMIC USE (J01)	ANTINEOPLASTIC AGENTS (L01)	Y	0.057
metformin	DRUGS USED IN DIABETES (A10)	ANTIHYPERTENSIVES (C02)	Y	0.056
brompheniramine	ANTIHISTAMINES FOR SYSTEMIC USE (R06)	CARDIAC THERAPY (C01)	Y	0.055
levamisole	ANTHELMINTICS (P02)	PSYCHOANALEPTICS (N06)	Y	0.054
altretamine	ANTINEOPLASTIC AGENTS (L01)	ANTIBACTERIALS FOR SYSTEMIC USE (J01)	Y	0.05
amodiaquine	ANTIPROTOZOALS (P01)	DIURETICS (C03)	Y	0.05
tacrine	PSYCHOANALEPTICS (N06)	ANTIBACTERIALS FOR SYSTEMIC USE (J01)	Y	0.05
trimethadione	ANTIEPILEPTICS (N03)	CARDIAC THERAPY (C01)	Y	0.049
mercaptopurine	ANTINEOPLASTIC AGENTS (L01)	PSYCHOLEPTICS (N05)	Y	0.047
ethosuximide	ANTIEPILEPTICS (N03)	ANTINEOPLASTIC AGENTS (L01)	Y	0.045
cyclopentolate	OPHTHALMOLOGICALS (S01)	ANTIEPILEPTICS (N03)	Y	0.044
ethosuximide	ANTIEPILEPTICS (N03)	CALCIUM CHANNEL BLOCKERS (C08)	Y	0.044
perhexiline	CALCIUM CHANNEL BLOCKERS (C08)	ANTIPROTOZOALS (P01)	Y	0.044
phenelzine	PSYCHOANALEPTICS (N06)	ANTIHYPERTENSIVES (C02)	Y	0.043
triamterene	DIURETICS (C03)	ANTINEOPLASTIC AGENTS (L01)	Y	0.041
flecainide	CARDIAC THERAPY (C01)	PSYCHOLEPTICS (N05)	Y	0.039
rolitetracycline	ANTIBACTERIALS FOR SYSTEMIC USE (J01)	PSYCHOANALEPTICS (N06)	Y	0.039
galantamine	PSYCHOANALEPTICS (N06)	CARDIAC THERAPY (C01)	Y	0.038
hydralazine	ANTIHYPERTENSIVES (C02)	OPHTHALMOLOGICALS (S01)	Y	0.036
metformin	DRUGS USED IN DIABETES (A10)	ANTIBACTERIALS FOR SYSTEMIC USE (J01)	Y	0.036
prazosin	ANTIHYPERTENSIVES (C02)	ANTIBACTERIALS FOR SYSTEMIC USE (J01)	Y	0.036
trimethadione	ANTIEPILEPTICS (N03)	OPHTHALMOLOGICALS (S01)	Y	0.036
amodiaquine	ANTIPROTOZOALS (P01)	ANTIBACTERIALS FOR SYSTEMIC USE (J01)	Y	0.035
cyclopentolate	OPHTHALMOLOGICALS (S01)	ANTIHISTAMINES FOR SYSTEMIC USE (R06)	Y	0.035
perhexiline	CALCIUM CHANNEL BLOCKERS (C08)	ANTHELMINTICS (P02)	Y	0.035
galantamine	PSYCHOANALEPTICS (N06)	DIURETICS (C03)	Y	0.033
atovaquone	ANTIPROTOZOALS (P01)	ANTHELMINTICS (P02)	Y	0.032
estradiol	SEX HORMONES AND MODULATORS OF THE GENITAL SYSTEM (G03)	CARDIAC THERAPY (C01)	Y	0.032
tacrine	PSYCHOANALEPTICS (N06)	ANTIINFLAMMATORY AND ANTIRHEUMATIC PRODUCTS (M01)	Y	0.031
trihexyphenidyl	ANTI-PARKINSON DRUGS (N04)	PSYCHOANALEPTICS (N06)	Y	0.031
prazosin	ANTIHYPERTENSIVES (C02)	PSYCHOANALEPTICS (N06)	Y	0.03
vorinostat	ANTINEOPLASTIC AGENTS (L01)	ANTHELMINTICS (P02)	Y	0.03
pioglitazone	DRUGS USED IN DIABETES (A10)	ANTIBACTERIALS FOR SYSTEMIC USE (J01)	Y	0.029
digoxin	CARDIAC THERAPY (C01)	ANTHELMINTICS (P02)	Y	0.028
doxazosin	ANTIHYPERTENSIVES (C02)	ANTIBACTERIALS FOR SYSTEMIC USE (J01)	Y	0.028

irinotecan	ANTINEOPLASTIC AGENTS (L01)	ANTIHYPERTENSIVES (C02)	Y	0.028
melatonin	PSYCHOLEPTICS (N05)	CARDIAC THERAPY (C01)	Y	0.028
sotalol	BETA BLOCKING AGENTS (C07)	ANTIBACTERIALS FOR SYSTEMIC USE (J01)	Y	0.028
paclitaxel	ANTINEOPLASTIC AGENTS (L01)	ANTHELMINTICS (P02)	Y	0.027
galantamine	PSYCHOANALEPTICS (N06)	PSYCHOANALEPTICS (N06)	N	0.027
prazosin	ANTIHYPERTENSIVES (C02)	ANTIPROTOZOALS (P01)	Y	0.026
gabapentin	ANTIEPILEPTICS (N03)	ANTIEPILEPTICS (N03)	N	0.026
diltiazem	CALCIUM CHANNEL BLOCKERS (C08)	DIURETICS (C03)	Y	0.024
imatinib	ANTINEOPLASTIC AGENTS (L01)	ANTINEOPLASTIC AGENTS (L01)	N	0.024
cyclopentolate	OPHTHALMOLOGICALS (S01)	ANTIBACTERIALS FOR SYSTEMIC USE (J01)	Y	0.023
proxymetacaine	OPHTHALMOLOGICALS (S01)	CARDIAC THERAPY (C01)	Y	0.023
verteporfin	OPHTHALMOLOGICALS (S01)	SEX HORMONES AND MODULATORS OF THE GENITAL SYSTEM (G03)	Y	0.023
diltiazem	CALCIUM CHANNEL BLOCKERS (C08)	CARDIAC THERAPY (C01)	Y	0.021
guanfacine	ANTIHYPERTENSIVES (C02)	PSYCHOLEPTICS (N05)	Y	0.021
halofantrine	ANTIPROTOZOALS (P01)	ANTIHYPERTENSIVES (C02)	Y	0.021
isocarboxazid	PSYCHOANALEPTICS (N06)	PSYCHOANALEPTICS (N06)	N	0.021
atovaquone	ANTIPROTOZOALS (P01)	PSYCHOANALEPTICS (N06)	Y	0.02
doxazosin	ANTIHYPERTENSIVES (C02)	ANTINEOPLASTIC AGENTS (L01)	Y	0.02
metformin	DRUGS USED IN DIABETES (A10)	ANTINEOPLASTIC AGENTS (L01)	Y	0.02
imatinib	ANTINEOPLASTIC AGENTS (L01)	ANTIHYPERTENSIVES (C02)	Y	0.019
raloxifene	SEX HORMONES AND MODULATORS OF THE GENITAL SYSTEM (G03)	ANTIBACTERIALS FOR SYSTEMIC USE (J01)	Y	0.019
trifluridine	OPHTHALMOLOGICALS (S01)	ANTIBACTERIALS FOR SYSTEMIC USE (J01)	Y	0.019
nalidixic_acid	ANTIBACTERIALS FOR SYSTEMIC USE (J01)	ANTIBACTERIALS FOR SYSTEMIC USE (J01)	N	0.019
diloxanide	ANTIPROTOZOALS (P01)	CARDIAC THERAPY (C01)	Y	0.018
selegiline	ANTI-PARKINSON DRUGS (N04)	ANTIHYPERTENSIVES (C02)	Y	0.017
trimethadione	ANTIEPILEPTICS (N03)	ANTIBACTERIALS FOR SYSTEMIC USE (J01)	Y	0.017
mephentermine	CARDIAC THERAPY (C01)	PSYCHOANALEPTICS (N06)	Y	0.016
procyclidine	ANTI-PARKINSON DRUGS (N04)	OPHTHALMOLOGICALS (S01)	Y	0.016
galantamine	PSYCHOANALEPTICS (N06)	ANTHELMINTICS (P02)	Y	0.015
irinotecan	ANTINEOPLASTIC AGENTS (L01)	ANTIBACTERIALS FOR SYSTEMIC USE (J01)	Y	0.015
melatonin	PSYCHOLEPTICS (N05)	SEX HORMONES AND MODULATORS OF THE GENITAL SYSTEM (G03)	Y	0.015
praziquantel	ANTHELMINTICS (P02)	PSYCHOLEPTICS (N05)	Y	0.015
tranylcypromine	PSYCHOANALEPTICS (N06)	ANTIHYPERTENSIVES (C02)	Y	0.015
trihexyphenidyl	ANTI-PARKINSON DRUGS (N04)	OPHTHALMOLOGICALS (S01)	Y	0.015
verteporfin	OPHTHALMOLOGICALS (S01)	ANTHELMINTICS (P02)	Y	0.015
vigabatrin	ANTIEPILEPTICS (N03)	PSYCHOANALEPTICS (N06)	Y	0.015
vinblastine	ANTINEOPLASTIC AGENTS (L01)	CARDIAC THERAPY (C01)	Y	0.015

amodiaquine	ANTIPROTOZOALS (P01)	CARDIAC THERAPY (C01)	Y	0.014
bepridil	CALCIUM CHANNEL BLOCKERS (C08)	PSYCHOLEPTICS (N05)	Y	0.014
mecamylamine	ANTIHYPERTENSIVES (C02)	ANTIBACTERIALS FOR SYSTEMIC USE (J01)	Y	0.014
mercaptopurine	ANTINEOPLASTIC AGENTS (L01)	OPHTHALMOLOGICALS (S01)	Y	0.014
milrinone	CARDIAC THERAPY (C01)	ANTIBACTERIALS FOR SYSTEMIC USE (J01)	Y	0.014
pargyline	ANTIHYPERTENSIVES (C02)	ANTI-PARKINSON DRUGS (N04)	Y	0.014
testosterone	SEX HORMONES AND MODULATORS OF THE GENITAL SYSTEM (G03)	CARDIAC THERAPY (C01)	Y	0.014
amoxapine	PSYCHOANALEPTICS (N06)	PSYCHOLEPTICS (N05)	Y	0.013
atovaquone	ANTIPROTOZOALS (P01)	ANTIBACTERIALS FOR SYSTEMIC USE (J01)	Y	0.013
bezafibrate	LIPID MODIFYING AGENTS (C10)	DRUGS USED IN DIABETES (A10)	Y	0.013
diltiazem	CALCIUM CHANNEL BLOCKERS (C08)	OPHTHALMOLOGICALS (S01)	Y	0.013
ethosuximide	ANTIEPILEPTICS (N03)	ANTIBACTERIALS FOR SYSTEMIC USE (J01)	Y	0.013
promazine	PSYCHOLEPTICS (N05)	PSYCHOANALEPTICS (N06)	Y	0.013
reserpine	ANTIHYPERTENSIVES (C02)	CARDIAC THERAPY (C01)	Y	0.013
vinblastine	ANTINEOPLASTIC AGENTS (L01)	ANTHELMINTICS (P02)	Y	0.013
trimethoprim	ANTIBACTERIALS FOR SYSTEMIC USE (J01)	ANTIBACTERIALS FOR SYSTEMIC USE (J01)	N	0.013
guanfacine	ANTIHYPERTENSIVES (C02)	ANTINEOPLASTIC AGENTS (L01)	Y	0.012
levamisole	ANTHELMINTICS (P02)	CARDIAC THERAPY (C01)	Y	0.012
mexiletine	CARDIAC THERAPY (C01)	BETA BLOCKING AGENTS (C07)	Y	0.012
milrinone	CARDIAC THERAPY (C01)	OPHTHALMOLOGICALS (S01)	Y	0.012
phenformin	DRUGS USED IN DIABETES (A10)	ANTHELMINTICS (P02)	Y	0.012
vorinostat	ANTINEOPLASTIC AGENTS (L01)	ANTIEPILEPTICS (N03)	Y	0.012
diltiazem	CALCIUM CHANNEL BLOCKERS (C08)	CALCIUM CHANNEL BLOCKERS (C08)	N	0.012
tolazamide	DRUGS USED IN DIABETES (A10)	ANTIBACTERIALS FOR SYSTEMIC USE (J01)	Y	0.011
tropicamide	OPHTHALMOLOGICALS (S01)	CARDIAC THERAPY (C01)	Y	0.011
mefloquine	ANTIPROTOZOALS (P01)	ANTINEOPLASTIC AGENTS (L01)	Y	0.01
mercaptopurine	ANTINEOPLASTIC AGENTS (L01)	ANTIINFLAMMATORY AND ANTIRHEUMATIC PRODUCTS (M01)	Y	0.01
vorinostat	ANTINEOPLASTIC AGENTS (L01)	CARDIAC THERAPY (C01)	Y	0.01
isocarboxazid	PSYCHOANALEPTICS (N06)	ANTI-PARKINSON DRUGS (N04)	Y	0.0098
bendroflumethiazide	DIURETICS (C03)	ANTIBACTERIALS FOR SYSTEMIC USE (J01)	Y	0.0097
procyclidine	ANTI-PARKINSON DRUGS (N04)	CARDIAC THERAPY (C01)	Y	0.0095
reserpine	ANTIHYPERTENSIVES (C02)	PSYCHOLEPTICS (N05)	Y	0.0095
mebendazole	ANTHELMINTICS (P02)	ANTIHISTAMINES FOR SYSTEMIC USE (R06)	Y	0.0094
midodrine	CARDIAC THERAPY (C01)	PSYCHOLEPTICS (N05)	Y	0.0094
diltiazem	CALCIUM CHANNEL BLOCKERS (C08)	ANTINEOPLASTIC AGENTS (L01)	Y	0.0092
etoposide	ANTINEOPLASTIC AGENTS (L01)	ANTIBACTERIALS FOR SYSTEMIC USE (J01)	Y	0.0091
amiodarone	CARDIAC THERAPY (C01)	CARDIAC THERAPY (C01)	N	0.0088

bepridil	CALCIUM CHANNEL BLOCKERS (C08)	ANTIPROTOZOALS (P01)	Y	0.0085
sotalol	BETA BLOCKING AGENTS (C07)	CARDIAC THERAPY (C01)	Y	0.0083
valproic_acid	ANTIEPILEPTICS (N03)	PSYCHOLEPTICS (N05)	Y	0.0083
biperiden	ANTI-PARKINSON DRUGS (N04)	ANTIBACTERIALS FOR SYSTEMIC USE (J01)	Y	0.0081
biperiden	ANTI-PARKINSON DRUGS (N04)	ANTIHISTAMINES FOR SYSTEMIC USE (R06)	Y	0.0081
levamisole	ANTHELMINTICS (P02)	ANTIBACTERIALS FOR SYSTEMIC USE (J01)	Y	0.0081
clomifene	SEX HORMONES AND MODULATORS OF THE GENITAL SYSTEM (G03)	ANTIBACTERIALS FOR SYSTEMIC USE (J01)	Y	0.0075
praziquantel	ANTHELMINTICS (P02)	PSYCHOANALEPTICS (N06)	Y	0.0075
prazosin	ANTIHYPERTENSIVES (C02)	CARDIAC THERAPY (C01)	Y	0.0075
promazine	PSYCHOLEPTICS (N05)	ANTIHISTAMINES FOR SYSTEMIC USE (R06)	Y	0.0075
primaquine	ANTIPROTOZOALS (P01)	CARDIAC THERAPY (C01)	Y	0.0074
primaquine	ANTIPROTOZOALS (P01)	ANTINEOPLASTIC AGENTS (L01)	Y	0.0074
remoxipride	PSYCHOLEPTICS (N05)	CARDIAC THERAPY (C01)	Y	0.0068
diloxanide	ANTIPROTOZOALS (P01)	ANTI-PARKINSON DRUGS (N04)	Y	0.0067
mercaptopurine	ANTINEOPLASTIC AGENTS (L01)	CARDIAC THERAPY (C01)	Y	0.0066
cyclopentolate	OPHTHALMOLOGICALS (S01)	PSYCHOANALEPTICS (N06)	Y	0.0065
amodiaquine	ANTIPROTOZOALS (P01)	PSYCHOLEPTICS (N05)	Y	0.0063
pergolide	ANTI-PARKINSON DRUGS (N04)	PSYCHOANALEPTICS (N06)	Y	0.0063
tolazamide	DRUGS USED IN DIABETES (A10)	PSYCHOLEPTICS (N05)	Y	0.0063
lymecycline	ANTIBACTERIALS FOR SYSTEMIC USE (J01)	PSYCHOANALEPTICS (N06)	Y	0.0062
propafenone	CARDIAC THERAPY (C01)	BETA BLOCKING AGENTS (C07)	Y	0.0062
amiodarone	CARDIAC THERAPY (C01)	CALCIUM CHANNEL BLOCKERS (C08)	Y	0.0059
buspirone	PSYCHOLEPTICS (N05)	BETA BLOCKING AGENTS (C07)	Y	0.0058
diloxanide	ANTIPROTOZOALS (P01)	ANTIEPILEPTICS (N03)	Y	0.0058
triamterene	DIURETICS (C03)	OPHTHALMOLOGICALS (S01)	Y	0.0058
trihexyphenidyl	ANTI-PARKINSON DRUGS (N04)	CARDIAC THERAPY (C01)	Y	0.0058
halofantrine	ANTIPROTOZOALS (P01)	PSYCHOLEPTICS (N05)	Y	0.0056
mephentermine	CARDIAC THERAPY (C01)	ANTIBACTERIALS FOR SYSTEMIC USE (J01)	Y	0.0056
quinidine	CARDIAC THERAPY (C01)	ANTIPROTOZOALS (P01)	Y	0.0056
rescinnamine	ANTIHYPERTENSIVES (C02)	PSYCHOLEPTICS (N05)	Y	0.0056
sulfadiazine	ANTIBACTERIALS FOR SYSTEMIC USE (J01)	ANTINEOPLASTIC AGENTS (L01)	Y	0.0056
biperiden	ANTI-PARKINSON DRUGS (N04)	OPHTHALMOLOGICALS (S01)	Y	0.0054
levamisole	ANTHELMINTICS (P02)	DIURETICS (C03)	Y	0.0054
memantine	PSYCHOANALEPTICS (N06)	DIURETICS (C03)	Y	0.0054
acetohexamide	DRUGS USED IN DIABETES (A10)	ANTIBACTERIALS FOR SYSTEMIC USE (J01)	Y	0.0053
diloxanide	ANTIPROTOZOALS (P01)	BETA BLOCKING AGENTS (C07)	Y	0.0048
diloxanide	ANTIPROTOZOALS (P01)	ANTIHYPERTENSIVES (C02)	Y	0.0048

spironolactone	DIURETICS (C03)	ANTIINFLAMMATORY AND ANTIRHEUMATIC PRODUCTS (M01)	Y	0.0048
gemfibrozil	LIPID MODIFYING AGENTS (C10)	ANTIBACTERIALS FOR SYSTEMIC USE (J01)	Y	0.0047
mebendazole	ANTHELMINTICS (P02)	ANTIBACTERIALS FOR SYSTEMIC USE (J01)	Y	0.0047
mefloquine	ANTIPROTOZOALS (P01)	CALCIUM CHANNEL BLOCKERS (C08)	Y	0.0047
minaprine	PSYCHOANALEPTICS (N06)	PSYCHOLEPTICS (N05)	Y	0.0047
phenformin	DRUGS USED IN DIABETES (A10)	ANTINEOPLASTIC AGENTS (L01)	Y	0.0047
risperidone	PSYCHOLEPTICS (N05)	PSYCHOANALEPTICS (N06)	Y	0.0047
albendazole	ANTHELMINTICS (P02)	ANTIHISTAMINES FOR SYSTEMIC USE (R06)	Y	0.0046
flecainide	CARDIAC THERAPY (C01)	ANTIEPILEPTICS (N03)	Y	0.0046
mecamylamine	ANTIHYPERTENSIVES (C02)	ANTINEOPLASTIC AGENTS (L01)	Y	0.0046
primaquine	ANTIPROTOZOALS (P01)	ANTIHISTAMINES FOR SYSTEMIC USE (R06)	Y	0.0046
biperiden	ANTI-PARKINSON DRUGS (N04)	CARDIAC THERAPY (C01)	Y	0.0045
carbamazepine	ANTIEPILEPTICS (N03)	ANTIEPILEPTICS (N03)	N	0.0044
estradiol	SEX HORMONES AND MODULATORS OF THE GENITAL SYSTEM (G03)	ANTIBACTERIALS FOR SYSTEMIC USE (J01)	Y	0.0038
melatonin	PSYCHOLEPTICS (N05)	ANTIEPILEPTICS (N03)	Y	0.0038
reserpine	ANTIHYPERTENSIVES (C02)	LIPID MODIFYING AGENTS (C10)	Y	0.0038
testosterone	SEX HORMONES AND MODULATORS OF THE GENITAL SYSTEM (G03)	ANTIINFLAMMATORY AND ANTIRHEUMATIC PRODUCTS (M01)	Y	0.0038
trihexyphenidyl	ANTI-PARKINSON DRUGS (N04)	ANTIHISTAMINES FOR SYSTEMIC USE (R06)	Y	0.0038
phenformin	DRUGS USED IN DIABETES (A10)	OPHTHALMOLOGICALS (S01)	Y	0.0037
phenformin	DRUGS USED IN DIABETES (A10)	ANTIHYPERTENSIVES (C02)	Y	0.0037
simvastatin	LIPID MODIFYING AGENTS (C10)	PSYCHOANALEPTICS (N06)	Y	0.0037
amodiaquine	ANTIPROTOZOALS (P01)	ANTINEOPLASTIC AGENTS (L01)	Y	0.0036
nitrofurantoin	ANTIBACTERIALS FOR SYSTEMIC USE (J01)	ANTIEPILEPTICS (N03)	Y	0.0036
pargyline	ANTIHYPERTENSIVES (C02)	ANTINEOPLASTIC AGENTS (L01)	Y	0.0036
acetohexamide	DRUGS USED IN DIABETES (A10)	PSYCHOLEPTICS (N05)	Y	0.0035
vorinostat	ANTINEOPLASTIC AGENTS (L01)	ANTIPROTOZOALS (P01)	Y	0.0035
fluvastatin	LIPID MODIFYING AGENTS (C10)	ANTINEOPLASTIC AGENTS (L01)	Y	0.003
galantamine	PSYCHOANALEPTICS (N06)	ANTI-PARKINSON DRUGS (N04)	Y	0.003
bupropion	PSYCHOANALEPTICS (N06)	ANTIHISTAMINES FOR SYSTEMIC USE (R06)	Y	0.0029
hydralazine	ANTIHYPERTENSIVES (C02)	ANTIBACTERIALS FOR SYSTEMIC USE (J01)	Y	0.0029
bepridil	CALCIUM CHANNEL BLOCKERS (C08)	BETA BLOCKING AGENTS (C07)	Y	0.0028
brinzolamide	OPHTHALMOLOGICALS (S01)	DIURETICS (C03)	Y	0.0028
cyproterone	SEX HORMONES AND MODULATORS OF THE GENITAL SYSTEM (G03)	ANTIBACTERIALS FOR SYSTEMIC USE (J01)	Y	0.0028
diltiazem	CALCIUM CHANNEL BLOCKERS (C08)	DRUGS USED IN DIABETES (A10)	Y	0.0028
ethosuximide	ANTIEPILEPTICS (N03)	OPHTHALMOLOGICALS (S01)	Y	0.0028
fluoxetine	PSYCHOANALEPTICS (N06)	PSYCHOLEPTICS (N05)	Y	0.0028
gabapentin	ANTIEPILEPTICS (N03)	PSYCHOANALEPTICS (N06)	Y	0.0028

mefloquine	ANTIPROTOZOALS (P01)	ANTIHISTAMINES FOR SYSTEMIC USE (R06)	Y	0.0028
perhexiline	CALCIUM CHANNEL BLOCKERS (C08)	CARDIAC THERAPY (C01)	Y	0.0028
procyclidine	ANTI-PARKINSON DRUGS (N04)	ANTIPROTOZOALS (P01)	Y	0.0028
risperidone	PSYCHOLEPTICS (N05)	ANTI-PARKINSON DRUGS (N04)	Y	0.0028
risperidone	PSYCHOLEPTICS (N05)	ANTIEPILEPTICS (N03)	Y	0.0028
tacrine	PSYCHOANALEPTICS (N06)	CARDIAC THERAPY (C01)	Y	0.0028
testosterone	SEX HORMONES AND MODULATORS OF THE GENITAL SYSTEM (G03)	ANTIBACTERIALS FOR SYSTEMIC USE (J01)	Y	0.0028
acetohexamide	DRUGS USED IN DIABETES (A10)	ANTINEOPLASTIC AGENTS (L01)	Y	0.0027
albendazole	ANTHELMINTICS (P02)	PSYCHOLEPTICS (N05)	Y	0.0027
brompheniramine	ANTIHISTAMINES FOR SYSTEMIC USE (R06)	PSYCHOANALEPTICS (N06)	Y	0.0027
tolazamide	DRUGS USED IN DIABETES (A10)	CARDIAC THERAPY (C01)	Y	0.0027
vorinostat	ANTINEOPLASTIC AGENTS (L01)	ANTIHISTAMINES FOR SYSTEMIC USE (R06)	Y	0.0026
amiodarone	CARDIAC THERAPY (C01)	ANTIHYPERTENSIVES (C02)	Y	0.002
amoxapine	PSYCHOANALEPTICS (N06)	CARDIAC THERAPY (C01)	Y	0.002
bendroflumethiazide	DIURETICS (C03)	OPHTHALMOLOGICALS (S01)	Y	0.0019
buspirone	PSYCHOLEPTICS (N05)	ANTIBACTERIALS FOR SYSTEMIC USE (J01)	Y	0.0019
cinoxacin	ANTIBACTERIALS FOR SYSTEMIC USE (J01)	PSYCHOANALEPTICS (N06)	Y	0.0019
clozapine	PSYCHOLEPTICS (N05)	ANTIBACTERIALS FOR SYSTEMIC USE (J01)	Y	0.0019
hydralazine	ANTIHYPERTENSIVES (C02)	ANTIPROTOZOALS (P01)	Y	0.0019
lovastatin	LIPID MODIFYING AGENTS (C10)	ANTINEOPLASTIC AGENTS (L01)	Y	0.0019
lovastatin	LIPID MODIFYING AGENTS (C10)	PSYCHOANALEPTICS (N06)	Y	0.0019
mebendazole	ANTHELMINTICS (P02)	PSYCHOLEPTICS (N05)	Y	0.0019
mercaptopurine	ANTINEOPLASTIC AGENTS (L01)	PSYCHOANALEPTICS (N06)	Y	0.0019
methazolamide	OPHTHALMOLOGICALS (S01)	PSYCHOANALEPTICS (N06)	Y	0.0019
metolazone	DIURETICS (C03)	ANTIBACTERIALS FOR SYSTEMIC USE (J01)	Y	0.0019
mifepristone	SEX HORMONES AND MODULATORS OF THE GENITAL SYSTEM (G03)	ANTHELMINTICS (P02)	Y	0.0019
paclitaxel	ANTINEOPLASTIC AGENTS (L01)	ANTIPROTOZOALS (P01)	Y	0.0019
procainamide	CARDIAC THERAPY (C01)	PSYCHOLEPTICS (N05)	Y	0.0019
pyrimethamine	ANTIPROTOZOALS (P01)	PSYCHOLEPTICS (N05)	Y	0.0019
risperidone	PSYCHOLEPTICS (N05)	ANTIBACTERIALS FOR SYSTEMIC USE (J01)	Y	0.0019
rolitetracycline	ANTIBACTERIALS FOR SYSTEMIC USE (J01)	OPHTHALMOLOGICALS (S01)	Y	0.0019
sulpiride	PSYCHOLEPTICS (N05)	DIURETICS (C03)	Y	0.0019
terfenadine	ANTIHISTAMINES FOR SYSTEMIC USE (R06)	CARDIAC THERAPY (C01)	Y	0.0019
triamterene	DIURETICS (C03)	ANTIPROTOZOALS (P01)	Y	0.0019
acetohexamide	DRUGS USED IN DIABETES (A10)	OPHTHALMOLOGICALS (S01)	Y	0.0018
brompheniramine	ANTIHISTAMINES FOR SYSTEMIC USE (R06)	ANTI-PARKINSON DRUGS (N04)	Y	0.0018
cyproterone	SEX HORMONES AND MODULATORS OF THE GENITAL SYSTEM (G03)	ANTIINFLAMMATORY AND ANTIRHEUMATIC PRODUCTS (M01)	Y	0.0018

danazol	SEX HORMONES AND MODULATORS OF THE GENITAL SYSTEM (G03)	ANTIBACTERIALS FOR SYSTEMIC USE (J01)	Y	0.0018
guanfacine	ANTIHYPERTENSIVES (C02)	CARDIAC THERAPY (C01)	Y	0.0018
levamisole	ANTHELMINTICS (P02)	BETA BLOCKING AGENTS (C07)	Y	0.0018
levamisole	ANTHELMINTICS (P02)	OPHTHALMOLOGICALS (S01)	Y	0.0018
memantine	PSYCHOANALEPTICS (N06)	CARDIAC THERAPY (C01)	Y	0.0018
metaraminol	CARDIAC THERAPY (C01)	ANTIBACTERIALS FOR SYSTEMIC USE (J01)	Y	0.0018
nomifensine	PSYCHOANALEPTICS (N06)	CARDIAC THERAPY (C01)	Y	0.0018
pergolide	ANTI-PARKINSON DRUGS (N04)	CARDIAC THERAPY (C01)	Y	0.0018
proxymetacaine	OPHTHALMOLOGICALS (S01)	ANTIHISTAMINES FOR SYSTEMIC USE (R06)	Y	0.0018
sulfapyridine	ANTIBACTERIALS FOR SYSTEMIC USE (J01)	ANTINEOPLASTIC AGENTS (L01)	Y	0.0018
trimethoprim	ANTIBACTERIALS FOR SYSTEMIC USE (J01)	OPHTHALMOLOGICALS (S01)	Y	0.0018
azacitidine	ANTINEOPLASTIC AGENTS (L01)	ANTIBACTERIALS FOR SYSTEMIC USE (J01)	Y	0.00099
fluvastatin	LIPID MODIFYING AGENTS (C10)	PSYCHOLEPTICS (N05)	Y	0.00099
galantamine	PSYCHOANALEPTICS (N06)	SEX HORMONES AND MODULATORS OF THE GENITAL SYSTEM (G03)	Y	0.00099
metformin	DRUGS USED IN DIABETES (A10)	PSYCHOLEPTICS (N05)	Y	0.00099
pioglitazone	DRUGS USED IN DIABETES (A10)	LIPID MODIFYING AGENTS (C10)	Y	0.00098
remoxipride	PSYCHOLEPTICS (N05)	ANTIBACTERIALS FOR SYSTEMIC USE (J01)	Y	0.00098
vinblastine	ANTINEOPLASTIC AGENTS (L01)	ANTIHISTAMINES FOR SYSTEMIC USE (R06)	Y	0.00098
hydrochlorothiazide	DIURETICS (C03)	OPHTHALMOLOGICALS (S01)	Y	0.00097
bupropion	PSYCHOANALEPTICS (N06)	ANTINEOPLASTIC AGENTS (L01)	Y	0.00096
diloxanide	ANTIPROTOZOALS (P01)	PSYCHOANALEPTICS (N06)	Y	0.00096
melatonin	PSYCHOLEPTICS (N05)	ANTINEOPLASTIC AGENTS (L01)	Y	0.00096
methoxamine	CARDIAC THERAPY (C01)	PSYCHOLEPTICS (N05)	Y	0.00096
methoxamine	CARDIAC THERAPY (C01)	PSYCHOANALEPTICS (N06)	Y	0.00096
paclitaxel	ANTINEOPLASTIC AGENTS (L01)	PSYCHOANALEPTICS (N06)	Y	0.00096
paclitaxel	ANTINEOPLASTIC AGENTS (L01)	ANTIINFLAMMATORY AND ANTIRHEUMATIC PRODUCTS (M01)	Y	0.00096
practolol	BETA BLOCKING AGENTS (C07)	CARDIAC THERAPY (C01)	Y	0.00096
tranylcypromine	PSYCHOANALEPTICS (N06)	CARDIAC THERAPY (C01)	Y	0.00096
atovaquone	ANTIPROTOZOALS (P01)	ANTI-PARKINSON DRUGS (N04)	Y	0.00095
atovaquone	ANTIPROTOZOALS (P01)	LIPID MODIFYING AGENTS (C10)	Y	0.00095
estradiol	SEX HORMONES AND MODULATORS OF THE GENITAL SYSTEM (G03)	PSYCHOLEPTICS (N05)	Y	0.00095
mefloquine	ANTIPROTOZOALS (P01)	ANTIBACTERIALS FOR SYSTEMIC USE (J01)	Y	0.00095
primidone	ANTIEPILEPTICS (N03)	PSYCHOLEPTICS (N05)	Y	0.00095
reserpine	ANTIHYPERTENSIVES (C02)	ANTIPROTOZOALS (P01)	Y	0.00095
reserpine	ANTIHYPERTENSIVES (C02)	ANTIEPILEPTICS (N03)	Y	0.00095
rolitetracycline	ANTIBACTERIALS FOR SYSTEMIC USE (J01)	ANTIEPILEPTICS (N03)	Y	0.00095
dopamine	CARDIAC THERAPY (C01)	CARDIAC THERAPY (C01)	N	0.00095

amiloride	DIURETICS (C03)	CARDIAC THERAPY (C01)	Y	0.00094
gemfibrozil	LIPID MODIFYING AGENTS (C10)	CARDIAC THERAPY (C01)	Y	0.00094
mebendazole	ANTHELMINTICS (P02)	OPHTHALMOLOGICALS (S01)	Y	0.00094
praziquantel	ANTHELMINTICS (P02)	CARDIAC THERAPY (C01)	Y	0.00094
praziquantel	ANTHELMINTICS (P02)	ANTINEOPLASTIC AGENTS (L01)	Y	0.00094
praziquantel	ANTHELMINTICS (P02)	ANTIHYPERTENSIVES (C02)	Y	0.00094
selegiline	ANTI-PARKINSON DRUGS (N04)	CARDIAC THERAPY (C01)	Y	0.00094
tacrine	PSYCHOANALEPTICS (N06)	OPHTHALMOLOGICALS (S01)	Y	0.00094
cyclopentolate	OPHTHALMOLOGICALS (S01)	CARDIAC THERAPY (C01)	Y	0.00093
disopyramide	CARDIAC THERAPY (C01)	OPHTHALMOLOGICALS (S01)	Y	0.00093
dorzolamide	OPHTHALMOLOGICALS (S01)	DIURETICS (C03)	Y	0.00093
dydrogesterone	SEX HORMONES AND MODULATORS OF THE GENITAL SYSTEM (G03)	OPHTHALMOLOGICALS (S01)	Y	0.00093
dydrogesterone	SEX HORMONES AND MODULATORS OF THE GENITAL SYSTEM (G03)	ANTHELMINTICS (P02)	Y	0.00093
lomustine	ANTINEOPLASTIC AGENTS (L01)	DRUGS USED IN DIABETES (A10)	Y	0.00093
lomustine	ANTINEOPLASTIC AGENTS (L01)	ANTIEPILEPTICS (N03)	Y	0.00093
perhexiline	CALCIUM CHANNEL BLOCKERS (C08)	PSYCHOANALEPTICS (N06)	Y	0.00093
phenformin	DRUGS USED IN DIABETES (A10)	ANTIBACTERIALS FOR SYSTEMIC USE (J01)	Y	0.00093
cyproterone	SEX HORMONES AND MODULATORS OF THE GENITAL SYSTEM (G03)	CARDIAC THERAPY (C01)	Y	0.00092
diltiazem	CALCIUM CHANNEL BLOCKERS (C08)	PSYCHOANALEPTICS (N06)	Y	0.00092
milrinone	CARDIAC THERAPY (C01)	ANTIINFLAMMATORY AND ANTIRHEUMATIC PRODUCTS (M01)	Y	0.00092
trimethadione	ANTIEPILEPTICS (N03)	ANTINEOPLASTIC AGENTS (L01)	Y	0.00092
tropicamide	OPHTHALMOLOGICALS (S01)	PSYCHOLEPTICS (N05)	Y	0.00092
tropicamide	OPHTHALMOLOGICALS (S01)	ANTIBACTERIALS FOR SYSTEMIC USE (J01)	Y	0.00092
valproic_acid	ANTIEPILEPTICS (N03)	PSYCHOANALEPTICS (N06)	Y	0.00092
gefitinib	ANTINEOPLASTIC AGENTS (L01)	ANTIPROTOZOALS (P01)	Y	0.00091
guanfacine	ANTIHYPERTENSIVES (C02)	OPHTHALMOLOGICALS (S01)	Y	0.00091
proxymetacaine	OPHTHALMOLOGICALS (S01)	PSYCHOANALEPTICS (N06)	Y	0.00091
trimethoprim	ANTIBACTERIALS FOR SYSTEMIC USE (J01)	CARDIAC THERAPY (C01)	Y	0.00091
amrinone	CARDIAC THERAPY (C01)	BETA BLOCKING AGENTS (C07)	Y	9.00E-04
amrinone	CARDIAC THERAPY (C01)	PSYCHOANALEPTICS (N06)	Y	9.00E-04
amrinone	CARDIAC THERAPY (C01)	ANTIBACTERIALS FOR SYSTEMIC USE (J01)	Y	9.00E-04
chloroquine	ANTIPROTOZOALS (P01)	PSYCHOLEPTICS (N05)	Y	9.00E-04
tolazamide	DRUGS USED IN DIABETES (A10)	ANTINEOPLASTIC AGENTS (L01)	Y	9.00E-04
memantine	PSYCHOANALEPTICS (N06)	PSYCHOANALEPTICS (N06)	N	9.00E-04
acetohexamide	DRUGS USED IN DIABETES (A10)	CALCIUM CHANNEL BLOCKERS (C08)	Y	0.00089
acetohexamide	DRUGS USED IN DIABETES (A10)	ANTIINFLAMMATORY AND ANTIRHEUMATIC PRODUCTS (M01)	Y	0.00089
amodiaquine	ANTIPROTOZOALS (P01)	ANTIHYPERTENSIVES (C02)	Y	0.00089

lymecycline	ANTIBACTERIALS FOR SYSTEMIC USE (J01)	ANTIPROTOZOALS (P01)	Y	0.00089
proguanil	ANTIPROTOZOALS (P01)	PSYCHOLEPTICS (N05)	Y	0.00089
propafenone	CARDIAC THERAPY (C01)	ANTIEPILEPTICS (N03)	Y	0.00089
brompheniramine	ANTIHISTAMINES FOR SYSTEMIC USE (R06)	PSYCHOLEPTICS (N05)	Y	0.00088
vorinostat	ANTINEOPLASTIC AGENTS (L01)	BETA BLOCKING AGENTS (C07)	Y	0.00087
vorinostat	ANTINEOPLASTIC AGENTS (L01)	SEX HORMONES AND MODULATORS OF THE GENITAL SYSTEM (G03)	Y	0.00087
hydroflumethiazide	DIURETICS (C03)	OPHTHALMOLOGICALS (S01)	Y	0.00086
